# A novel role of the soybean clock gene *LUX ARRHYTHMO* in male reproductive development

**DOI:** 10.1038/s41598-017-10823-y

**Published:** 2017-09-06

**Authors:** Lim Chee Liew, Mohan B. Singh, Prem L. Bhalla

**Affiliations:** 1 Plant Molecular Biology and Biotechnology Laboratory, Faculty of Veterinary and Agricultural Sciences, The University of Melbourne, Parkville, Victoria 3010 Australia; 2 Present Address: Department of Animal, Plant and Soil Science, School of Life Science, La Trobe University, Bundoora, VIC 3086 Australia

## Abstract

The evening complex of ELF4-ELF3-LUX proteins is an integral component of a plant circadian clock. *LUX ARRHYTHMO* (*LUX*) is one of the key components of the evening complex, and that play a key role in circadian rhythms and flowering. Here, we report that diverged soybean *LUX* has the additional role in male reproductive development. We studied diurnal and circadian rhythms of soybean *LUX* (*GmLUXa*, *GmLUXb*, and *GmLUXc)* using qRT-PCR, and show its nuclear localisation by particle bombardment. Yeast-two hybrid (Y2H) studies indicate that both GmLUXb and GmLUXc form an evening complex with GmELF4b and GmELF3a, respectively. Ectopic expression of *GmLUXb* in *Arabidopsis lux* mutants can complement functions of *AtLUX*, whereas *GmLUXc* generates novel phenotypes of serrated leaves, stunted plants, shortened anther filament, and low seed set. Overall, our results suggest that the *LUX* gene has diverged in soybean where *GmLUXb* and *GmLUXc* share the role to control flowering time, but *GmLUXc* has evolved to regulate anther filament growth and seed set by regulating the Gibberellin hormone biosynthesis pathway.

## Introduction

The world’s population is projected to surpass 9 billion by 2050; feeding this enormous population requires a doubling of food production from the same amount of arable land. Moreover, looming climate change threatens our food security, as plant reproductive development like flowering and grain production are inherently sensitive to the changes in the external environment. Unlike animals, plants are sessile; it is important for plants to integrate exogenous signals with endogenous rhythms to ensure the best time for progression to reproductive stage to maximise reproductive success^1, 2^. The circadian clock system is well known for its role in synchronising environmental signals with endogenous rhythms to ensure proper timing of flowering. Most of our knowledge on circadian clocks and flowering comes from a model plant, Arabidopsis^3^. Our knowledge of molecular components of the circadian clock system controlling flowering of food legume crops is lacking^4^. Food legume crops such as soybean, chickpea, faba bean, pea, and lentils are important for food and feed usage around the world. Soybean is a major food crops important for its seed oil and protein content. Being a legume, soybean forms symbioses with *Rhizobium* bacteria to fix amtmospheric nitrogen. The palaeopolyploid genome of soybean is results of multiple rounds of genome wide duplication^5, 6^. These duplications of the genome may result in pseudogenization, subfunctionalization, or neofunctionalization of genes. Among the flowering genes characterised in soybean, two of the main photoperiod responsive genes - *CONSTANS* (*CO*) and *FLOWERING LOCUS T* (*FT*) – display more than one orthologue with the functions in flowering time control. *CO* regulated by the circadian clock perceives and integrates environmental and endogenous signals to activate florigen (*FT*), to evoke floral transition. Four putative soybean *CO* orthologues were found, but only *GmCOL5* showed function in flowering time control^7^. Soybean genome includes 10 *FT*-like genes consisting of five homeologous pairs (*GmFT1*-*GmFT5*)^8^. Of the 10 *FT*-like genes, the transcripts levels of *GmFT2a* and *GmFT5a* are up-regulated by short-day and were shown to have a strong correlation with flowering, whereas their homeologues *GmFT2b* and *GmFT5b* showed no transcript in short-day-grown plants^8, 9^.

Soybean is well known for the discovery of photoperiodism^10^. Most commercial soybean cultivars have strong photoperiod requirements and are classified as maturity groups with a restricted latitudinal range. However, molecular basis underpinning maturity grouping remains unknown. In soybean, nine loci (*E1* to *E8*, and *J*) are associated with photoperiod and maturity. Positional cloning and candidate gene approaches have identified that *E1* encodes a B3 superfamily member^11, 12^, *E2* encodes an ortholog of *Arabidopsis* circadian clock component GI^11, 13^, and *E3* and *E4* encode the photoreceptors GmPHYA3 and GmPHYA2, respectively^14^. Also, there has been an attempt to associate cryptochromes, that is, blue light receptors, to explain the latitudinal distribution of soybean^15^.

Being sessile, plants sense and adjust to the environment changes by a circadian clock. The circadian clocks of plants have three main components: inputs, circadian oscillators, and outputs. External cues for example changes in light and temperature are conveyed to the circadian oscillator by the input pathway, which links to various output processes. Our understanding of the plant circadian clock is mostly based on studies using the model plant Arabidopsis. The core circadian clock of Arabidopsis comprises genes interacting via multiple transcriptional and post-transcriptional feedback loops to generate rhythmic gene expression. The morning loop consists of two PSEUDO RESPONSE REGULATOR (PRR) 7 and 9, LATE ELONGATED HYPOCOTYL (LHY), CIRCADIAN CLOCK-ASSOCIATED 1 (CCA1), and TIMING OF CAB EXPRESSION 1 (TOC1)) form the core loop^16^, while the evening loop is a complex of ELF3, ELF4, and LUX^17^. The circadian clock regulates multiple output pathways including growth, flowering,, photosynthesis, starch metabolism, and disease resistance^18^. It is well established that the evening complex (ELF3-ELF4-LUX) plays a vital role in maintaining circadian rhythms and synchronising growth and development^19^. Further, regulated growth controlled by the evening complex occurs by repression of the gene expression and activity of PIF4. LUX suppress its target gene via recruitment of the evening complex to their promoters^20^. However, it is not clear how the evening complex connects with other pathways to regulate plant development and physiology. Recent studies on Arabidopsis showed that plants have tissue-specific clocks, and evening complex components have tissue-specific expression patterns, but their functional significance is unknown. Moreover, preferential retention of circadian clock components during genome duplication events indicates positive selection^21^. The conserved function of the evening complex in crop plants suggests its vital role in crop adaptation to various environments. However, the molecular workings of the components of the clock and their wiring in crop plants still remains an open question in plant biology. Hence, molecular understanding of clock function in crop plants will pave the way to manipulate internal clocks for crop productivity in changing climates.

In this study, we characterise a soybean circadian clock gene in the evening complex, *LUX ARRHYTHMO*, *GmLUXb*, and *GmLUXc* and demonstrate conserved protein sequences, gene expression patterns, and subcellular localisation similar to Arabidopsis. The protein-protein analysis shows both GmLUXb and GmLUXc are able to forms an evening complex with GmELF3a and GmELF4b, suggesting that both of them have a role in the circadian clock of soybean. Complementation analysis of the *Arabidopsis lux-4* mutant with *GmLUXb* restores the phenotype back to wild-type, whereas *GmLUXc* introduces novel phenotypes including serrated leaves, compact and stunted inflorescence, shortened filament, and low seed set, suggesting neofunctionalization of *GmLUXc*. Further studies on *GmLUXc* functions suggest that it affects MYBs (*MYB21*, *MYB24*) and GA biosynthesis gene expression (*GA2ox1*, *GA20ox2*). Treatment with exogenous GA rescues the seed set of *GmLUXc* transgenic plants. This indicates that the GmLUXc drives anther filament elongation control and seed set by regulating the GA hormone biosynthesis pathway.

## Results

### Soybean homologues of *LUX ARRHYTHMO* shows conserved protein sequences, gene expression patterns, and subcellular localisation

**Figure 1:**
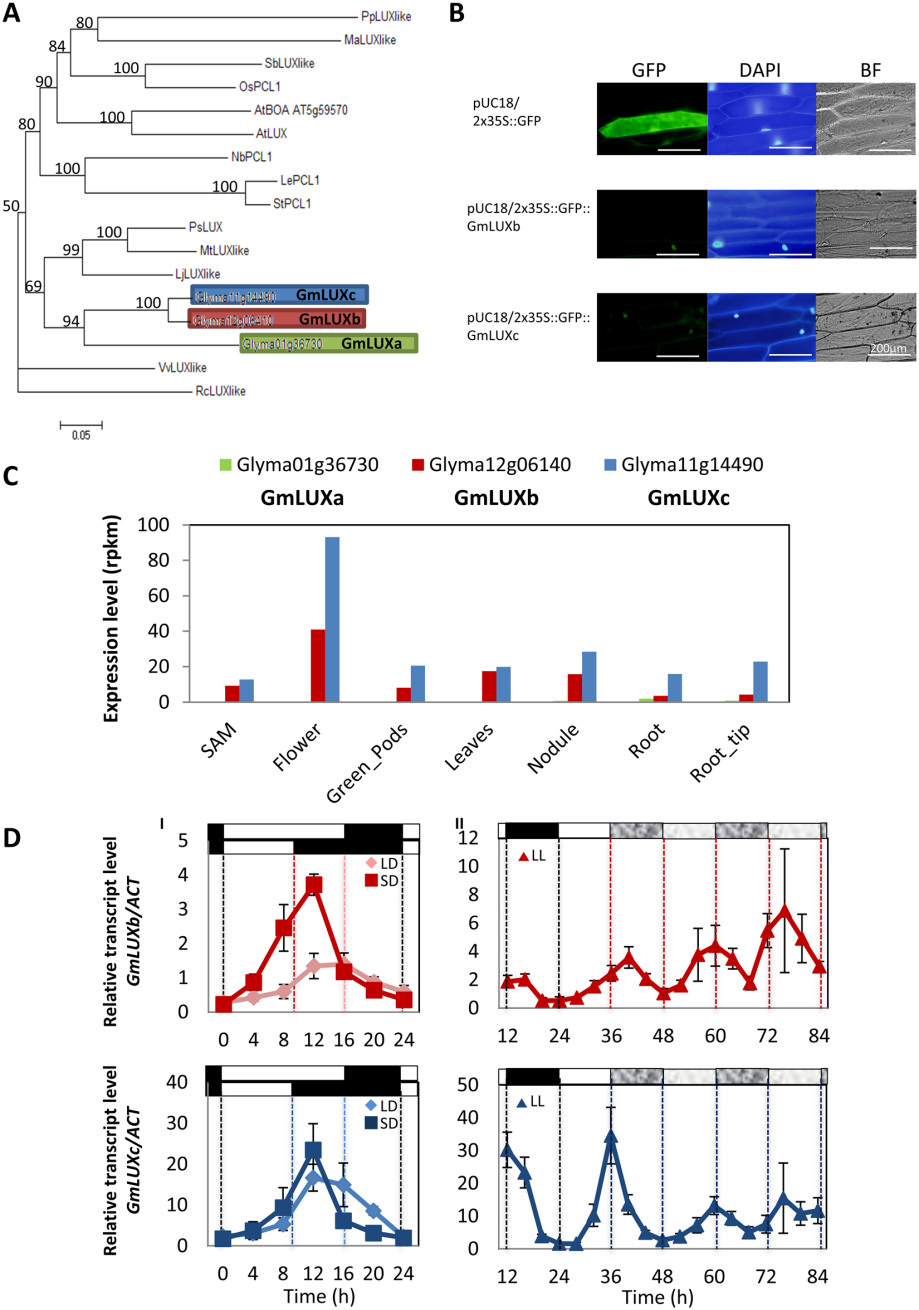
Characterisation of soybean *LUX ARRHYTHMO* (*LUX*). **(A)** Phylogenetic analysis of soybean LUX proteins. Three soybean homologues were identified in the soybean genome and designated as GmLUXa, GmLUXb, and GmLUXc. The tree is drawn to given scale, and the bootstrap values are shown at each node. **(B)** Sub-cellular localisation of GmLUXb and GmLUXc proteins in onion epidermal cells. Onion epidermal peels were bombarded with constructs 35S-GFP alone or 35S-GFP fused with either GmLUXb or GmLUXc sequences as described in methods and materials section. When GFP was expressed alone, the green fluorescence was dispersed throughout the cell, while green fluorescence from GFP-LUX fusion proteins were localized in nuclei. GFP: green fluorescence, DAPI: stained with 4′,6-diamidino-2-phenylindole dihydrochloride (DAPI), BF: under bright field. **(C)** Homologues of soybean *LUX* gene show tissue-specific expression. Expression of *GmLUXa*, *GmLUXb*, and *GmLUXc* in shoot apical meristem (SAM), flowers, green pods, leaves, nodules, root, and root tips. **(D)** Homologues of soybean *LUX* gene show diurnal and circadian rhythms (i) Diurnal rhythms of *GmLUXb* and *GmLUXc* under long-day (LD; 16 h light, 8 h dark) or short-day (SD; 10 h light, 14 h dark) conditions. (ii) Circadian rhythms of *GmLUXb* and *GmLUXc* under constant light (LL; 12 h light:12 h dark then continuous light). All plants were three weeks old at the time of sampling. Data are mean ± SE for n = three biological replicates, each consisting of pooled material from two plants. Day and night periods are depicted above the graph by open and closed bars, respectively.

Three soybean homologues of *LUX ARRHTHMO* were identified in a soybean genome sequence database; they are designated as *GmLUXa*, *GmLUXb*, and *GmLUXc*. Phylogenetic analysis (Fig. 1A) showed that these three soybean homologues clustered together and fall into the same clade with other legume (i.e., pea, medicago, and lotus) LUX proteins. Protein alignment revealed conserved amino acid sequences, especially on the DNA-binding MYB domain, except GmLUXa was truncated in the middle of the MYB domain, likely resulting in shortened protein than GmLUXb and GmLUXc (Supplementary Figure 1).

In *Arabidopsis*, *LUX* encodes a nuclear localised MYB domain protein with transcription factor activity^22, 23^. To study GmLUXb and GmLUXc subcellular localisation, we used transient assays where the epidermal cell layer of onion was bombarded with particles coated with DNA (*GmLUXb::GFP* and *GmLUXc::GFP*). The fluorescence was located in the nucleus of bombarded epidermal cells, indicating GmLUXb::GFP and GmLUXc::GFP are localised in the nucleus (Fig. 1B).

Tissue-specific expression study showed that *GmLUXa* was hardly expressed in any tissues examined, whereas highest expression of *GmLUXb* and *GmLUXc* were found in floral tissues (Fig. 1C). The expression level of *GmLUXc* in the flower was almost double of *GmLUXb* (Fig. 1C). Interestingly, *GmLUXc* has higher transcript level in all the tissue examined when compared to *GmLUXb*, especially at root and root tip where *GmLUXb* expression is low (Fig. 1C). Diurnal and circadian rhythms of *GmLUXb* and *GmLUXc* were further examined in soybean (Fig. 1D). Under long-day (LD) and short-day (SD) entrainment, both *GmLUXb* and *GmLUXc* transcripts showed clear diurnal rhythms where both genes peaked in the evening in both LD (ZT12-16) and SD (ZT12) conditions (Fig. 1D). Consistently, one evening element (AAAATATCT) was found at the promoter of *GmLUXb* and *GmLUXc*, respectively, at 461 bp and 480 bp upstream of the translational start site (Supplementary Table 1). Interestingly, only *GmLUXc* has a LUX binding site (LBS) as *AtLUX* but not in *GmLUXb* (Supplementary Table 1), which is 321 bp upstream of the translational start site for *GmLUXc* and 545 bp upstream of *AtLUX*. Distinct circadian rhythms were found between *GmLUXb* and *GmLUXc* under constant light (LL), showing *GmLUXb* transcripts gradually increased its level after released into LL condition, whereas *GmLUXc* displayed damped expression rhythms in LL (Fig. 1D). Previous study showed that LBS at *AtLUX* promoter was bound by *AtLUX in vivo* and ChIP assay further confirmed this result^20^. This binding account for self-regulation of gene expression of *AtLUX* by negative autoregulatory feedback loop. This may explain the difference of expression in LL between *GmLUXb* and *GmLUXc* where *GmLUXc* is damped after transferred to LL (Fig. 1D).

### Both GmLUXb and GmLUXc forms evening complex with GmELF4b and GmELF3a

In *Arabidopsis*, EARLY FLOWERING 4 (ELF4), EARLY FLOWERING 3 (ELF3), and LUX proteins are known to form an evening complex (EC) that transcriptionally regulates other clock genes and output genes in the evening^17^. Here, we isolated soybean homologues of *ELF3* and *ELF4*, designated as *GmELF4a* (Glyma11g35270), *GmELF4b* (Glyma18g03130), *GmELF3a* (Glyma04g05280), and *GmELF3b* (Glyma14g10530) (Supplementary Figures 2 and 3). Supplementary Figure 2 shows that both paralogous soybean pairs of *ELF3* and *ELF4* are highly conserved in protein sequences with other plant species. We also characterised diurnal and circadian rhythms of soybean *ELF3* and *ELF4* homologoues (Supplementary Figure 3). *GmELF4a* peaked at ZT8 in SD and ZT12 in LD, while *GmELF4b* peaked at ZT12 in SD and ZT12-16 in LD (Supplementary Figure 3A). On the other hand, both *GmELF3a* and *GmELF3b* peaked at ZT12 in SD and ZT12-16 in LD (Supplementary Figure 3B). Under the constant condition, like *GmLUX*, both *GmELF4* and *GmELF3* paralogous pairs showed different circadian rhythms, where *GmELF4a* and *GmELF3a* seemed to damp rapidly to a low level (Supplementary Figure 3). GmELF4b and GmELF3b maintained the transcript level after being subjected to continuous light (Supplementary Figure 3).

**Figure 2:**
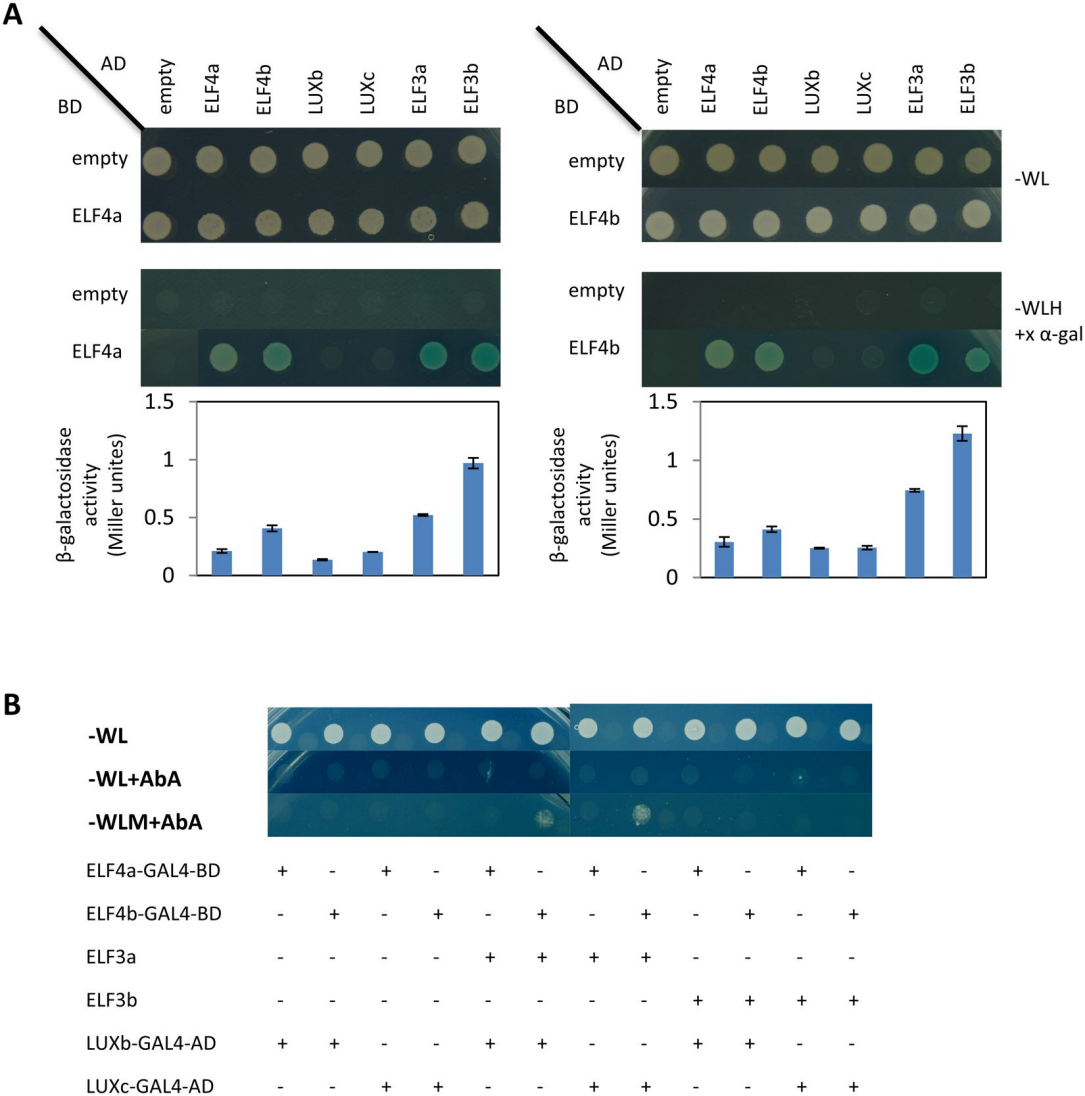
GmLUXb and GmLUXc interact with GmELF4b and GmELF3a in yeast. **(A)** Yeast-two hybrid assay between ELF4a/4b and each of ELF4a, ELF4b, LUXb, LUXc, ELF3a, and ELF3b. These experiments were repeated twice. The results were further confirmed by β-galactosidase activity; data are mean ± SE for n = 3. BD: binding domain, AD: activating domain. **(B)** Yeast-three hybrid assay between different combinations of ELF4a, ELF4b, LUXb, LUXc, ELF3a, and ELF3b. These experiments were repeated twice. Tryptophan (W), leucine (L), histidine (H), methionine (M), and AbA (Aureobasidin A).

We used yeast-two and yeast-three hybrids to examine protein-protein interactions of soybean ELF4, ELF3, and LUX. The yeast-two hybrid showed that both ELF4a and ELF4b interacted with ELF3a and ELF3b (Fig. 2A). Both ELF4a and ELF4b showed self-interaction and interaction with each other, whereas neither of ELF4a or ELF4b interacted with LUXb or LUXc (Fig. 2A). This observation was validated by quantitation of β-galactosidase activities (Fig. 2A). Using the yeast-three hybrid assay in combination with fusion proteins of ELF4a/ELF4b-GAL4-DNA binding domain (BD) and LUXb/LUXc-GAL4-activating domain (AD), we further confirmed that ELF4a/ELF4b could not interact independently with LUXb/c (Fig. 2B). Yeast growth was only observed in selection media (SD-WLM + AbA) in two combinations: (1) ELF4b-ELF3a-LUXb; (2) ELF4b-ELF3a-LUXc (Fig. 2B). These findings suggest two potential combinations of evening complexes in soybean, which include ELF4b and ELF3a and interact with either LUXb or LUXc.

### Ectopic expression of *EARLY FLOWERING 3 (ELF3)* and *EARLY FLOWERING 4 (ELF4)* in Arabidopsis

To further explore the functional divergence of *GmELF4* and *GmELF3*, ectopic expression of the *GmELF4* and *GmELF3* paralogous pair in *Arabidopsis elf4-1* and *elf3-1* mutants were carried out to check if soybean homologs can complement the function of *Arabidopsis* genes.

**Figure 3:**
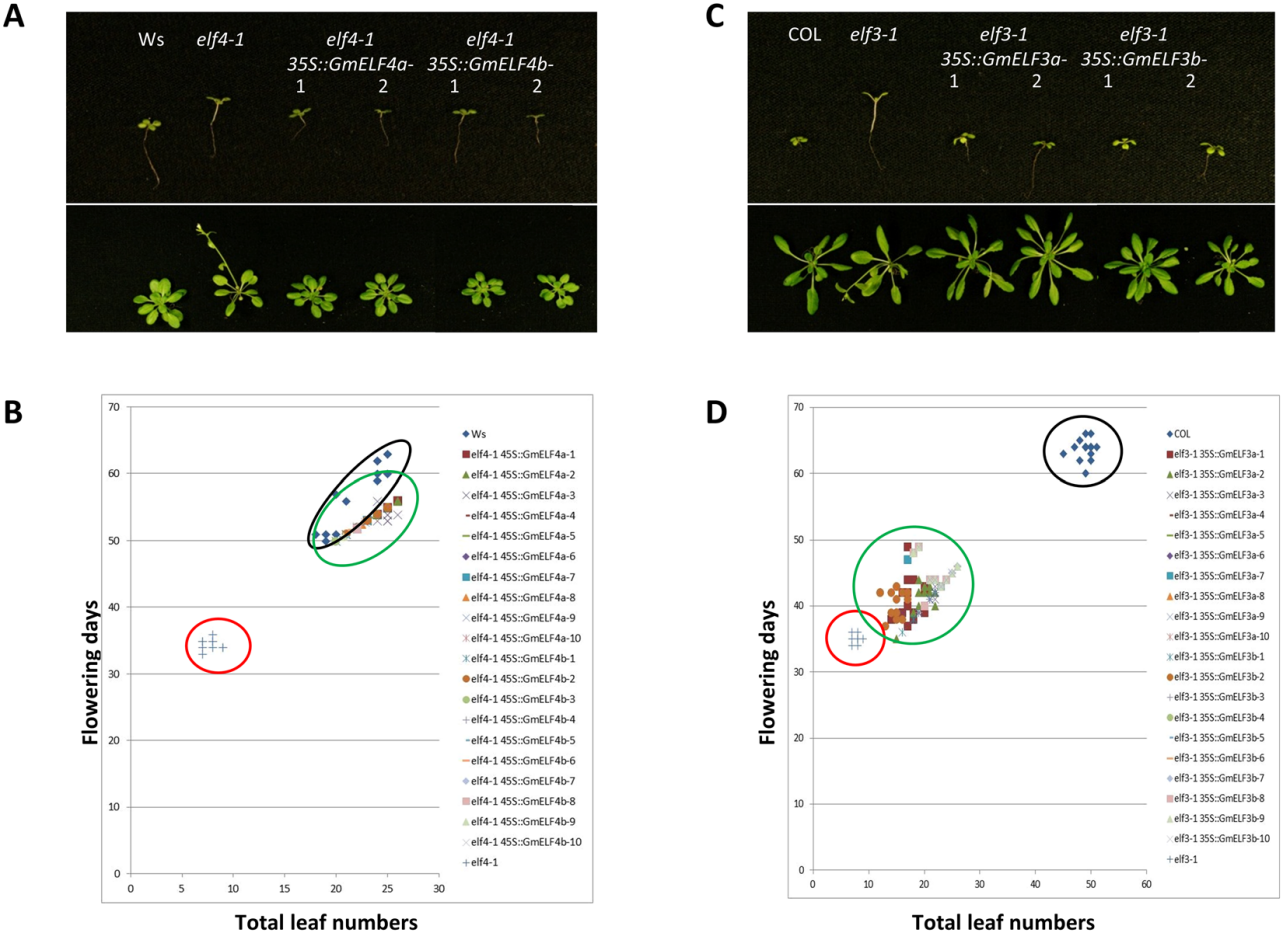
Ectopic expression of *GmELF4* homologs fully complements Arabidopsis mutant *elf4-1* hypocotyl and flowering time defects whereas *GmELF3 homologs* only partially rescue Arabidopsis *elf3-1* mutant. **(A)** Upper panel showing rescued elongated hypocotyl phenotype after ectopic expression of *GmELF4a* and *GmELF4b*; bottom panel showing rescued early flowering phenotype after ectopic expression of *GmELF4a* and *GmELF4b*. **(B)** Scatter plot of flowering time measurement in SD of wild-type (*Ws*), *elf4-1* mutant and transgenic plants overexpressing *GmELF4a* and *GmELF4b*. black circle: wild-type, red circle: mutant, green circle: transgene lines. Representative plants grown under SD conditions (10 L:14D) are shown. **(C)** Upper panel showing rescue of elongated hypocotyl phenotype after ectopic expression of *GmELF3a* and *GmELF3b*; bottom panel showing partially rescued early flowering phenotype after ectopic expression of *GmELF3a* and *GmELF3b*. **(D)** Scatter plot of flowering time measurement in SD of wild-type (*Col*), *elf3-1* mutant and transgenic plants overexpressing *GmELF3a* and *GmELF3b*. black circle: wild-type, red circle: mutant, green circle: transgene lines. Representative plants grown under SD conditions (10 L:14D) are shown.

The *Arabidopsis elf4-1* (Ws) mutant was sourced from the Feldmann T-DNA insertion mutant population, resulting in plants flowering early in both LD and SD with long hypocotyls and petioles^24^. Overexpression of *GmELF4a* or *GmELF4b* under a CaMV 35S promoter complemented the *elf4-1 Arabidopsis* mutant under SD conditions, rescuing the early-flowering phenotype of *elf4-1* to a flowering time similar to wild-type Ws (Fig. 3A,B). Also, the hypocotyl and petioles lengths of plants overexpressing *GmELF4a* or *GmELF4b* were greatly reduced when compared to the *elf4-1* mutant (Fig. 3A). These results suggest that *GmELF4a* and *GmELF4b* can complement *ELF4* functions in *Arabidopsis*.

The *Arabidopsis elf3-1* (Col) mutant was originally isolated as a photoperiod-insensitive early-flowering mutant also showed a long-hypocotyl phenotype^25^. Both over-expressions of *GmELF3a* or *GmELF3b* under the CaMV 35S promoter do not fully complement the *elf3-1 Arabidopsis* mutant under SD conditions, resulting in transgenic plants with flowering time between *elf3-1* mutant and wild-type COL and closer to *elf3-1* mutant (Fig. 3C,D). However, both overexpression transgenic plants of *GmELF3a* or *GmELF3b* rescued the long-hypocotyl phenotype of the *elf3-1* mutant (Fig. 3C). These results point out that neither overexpressing of *GmELF3a* nor *GmELF3b* can complement the loss-of-function in the *elf3-1* mutant, suggesting functional divergence of soybean and *Arabidopsis ELF3* and the complexity of the circadian clock system in the paleopolyploid species soybean.

### *GmLUXb* can complement the *Arabidopsis lux-4* mutant

**Figure 4:**
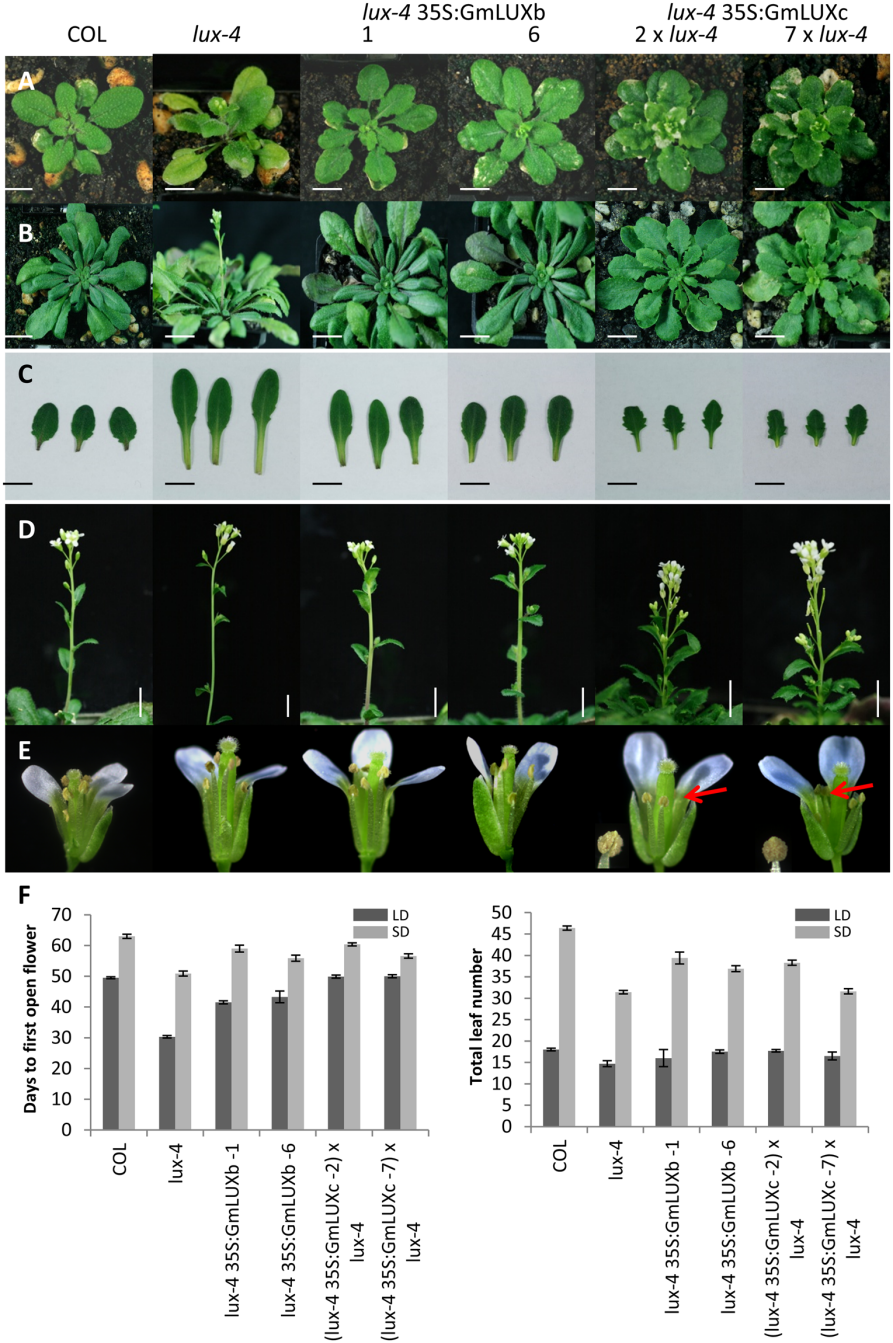
Ectopic expression of *GmLUXb* and *GmLUXc* in *Arabidopsis lux-4* mutant shows different phenotypes. **(A)** Representative plants are grown in LD (16 L: 8D). **(B)** Representative plants are grown in SD (10 L:14D). **(C)** Leave morphology of plants grown in SD, showing serrated leaves in *GmLUXc* overexpression transgenic plants. **(D)** Shoot architecture of *GmLUXc* plants grown in SD showing compact and stunted plants. **(E)** Fully opened flower from *GmLUXc* transgenic plants grown in SD showing shortened filament length (red arrows) but normal anther dehiscence. **(F)** Flowering time of *GmLUXb* and *GmLUXc* in *lux-4* mutant plants grown in LD and SD showed delayed flowering time; data are mean ± SE for n = 15–20. Scale bar =1 cm.

To study if *GmLUXb* and/or *GmLUXc* carry out the same functions as *Arabidopsis LUX* in flowering time control, a complementation analysis in the *lux-4* mutant was performed. The *Arabidopsis lux-4* mutant carries a nonsense mutation and produces a protein truncated right at the start of the MYB domain, generating in plants that flower early in both LD and SD with long hypocotyls and petioles^22, 23^. In *Arabidopsis*, overexpression of *AtLUX* in the *lux-4 Arabidopsis* mutant restored the circadian rhythms and hypocotyl growth^20^. Overexpression of *GmLUXb* under the CaMV 35S promoter complemented the *lux-4 Arabidopsis* mutant under LD (Fig. 4A) and SD (Fig. 4B) conditions, rescuing the early-flowering phenotype of *lux-4* to a flowering time similar to wild-type COL (Fig. 4F). In addition, the petioles lengths of plants overexpressing *GmLUXb* were greatly reduced when compared to the *lux-4* mutant (Fig. 4C), and weak inflorescence stem of the *lux-4* mutant were not found in plants overexpressing *GmLUXb* (Fig. 4D). Similar phenotypes were observed for overexpressing *GmLUXb* in COL wild-type plants (Supplementary Figure 4).

### Ectopic expression of *GmLUXc* in both the *Arabidopsis lux-4* mutant and COL wild-type show serrated leaves, compact and stunted plants, shortened anther filament, and low seed set

**Figure 5:**
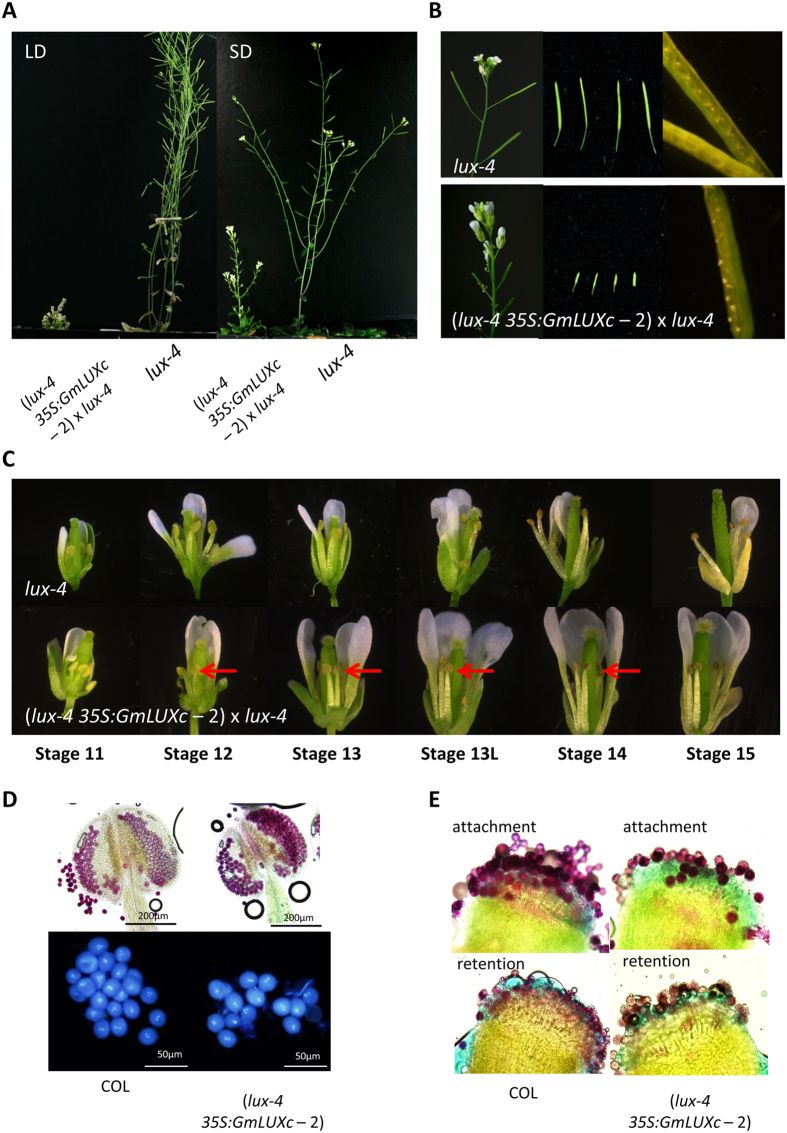
Ectopic expression of *GmLUXc* in *Arabidopsis lux-4* mutant shows novel phenotypes. **(A)** Representative plants grown in LD (16 L: 8D) and SD (10 L: 14D), showed compact and stunted plants of *lux-4 35S::GmLUXc*. (**B)** *lux-4 35S::GmLUXc* plants have compact inflorescence, short siliques, and low seed set as compared to *lux-4* mutant. **(C)** Flowers of *lux-4 and lux-4 35S::GmLUXc* at different stages of flower development (stage 11–15), showing shortened filament (red arrows) during all the developmental stages. **(D)** Alexander’s staining and DAPI staining of anthers and pollen of *Columbia* (*Col*) and *lux-4 35S::GmLUXc* plants, showing normal pollen. **(E)** Pollen attachment and retention assay of *Columbia* (*Col*) and *lux-4 35S::GmLUXc* plants, showing impaired pollen attachment of *lux-4 35S::GmLUXc* plants.

In contrast to the overexpression of *GmLUXb*, overexpression of *GmLUXc* in both *lux-4* mutants and COL wild-type delayed the early-flowering phenotype of *lux-4* but also introduced a novel phenotype that was not seen in either *lux-4* or *lux-4 35*S*::GmLUXb* plants (Fig. 4, Supplementary Figure 4). The first observation is serrated rosette and cauline leaves in both LD and SD-grown plants (Fig. 4A–C, Supplementary Figure 4). The transgenic plants also produced compact and stunted plants (Fig. 4D) where this phenotype was more severe in the LD condition (Fig. 5A). The T1 *lux-4 35S::GmLUXc* plants were sterile and developed short siliques with aborted seeds (Fig. 5B). Hand pollination of *lux-4 35S::GmLUXc* carpels with *lux-4* pollens yielded viable seed but not vice versa, suggesting the female fertility was normal. Observation of anthers on fully opened flowers showed that plants overexpressing *GmLUXc* have shorter filament length, but anthers dehisce and release pollens normally (Fig. 4E). A detailed analysis of flower development stages as assigned by Smyth *et al*.^26^ was performed (Fig. 5C). Figure 5C shows that filaments of plants overexpressing *GmLUXc* never elongated to the same level, as the stigma and stigmatic papillae did not mature as seen in *lux-4* plants. The phenotype of shortened filaments in *lux-4 35S::GmLUXc* plants could explain why self-pollination resulted in short siliques with low to nil seed set but could not account for reduced seed set by hand pollinating *35S::GmLUXc* pollen to *lux-4* carpels.

**Figure 6:**
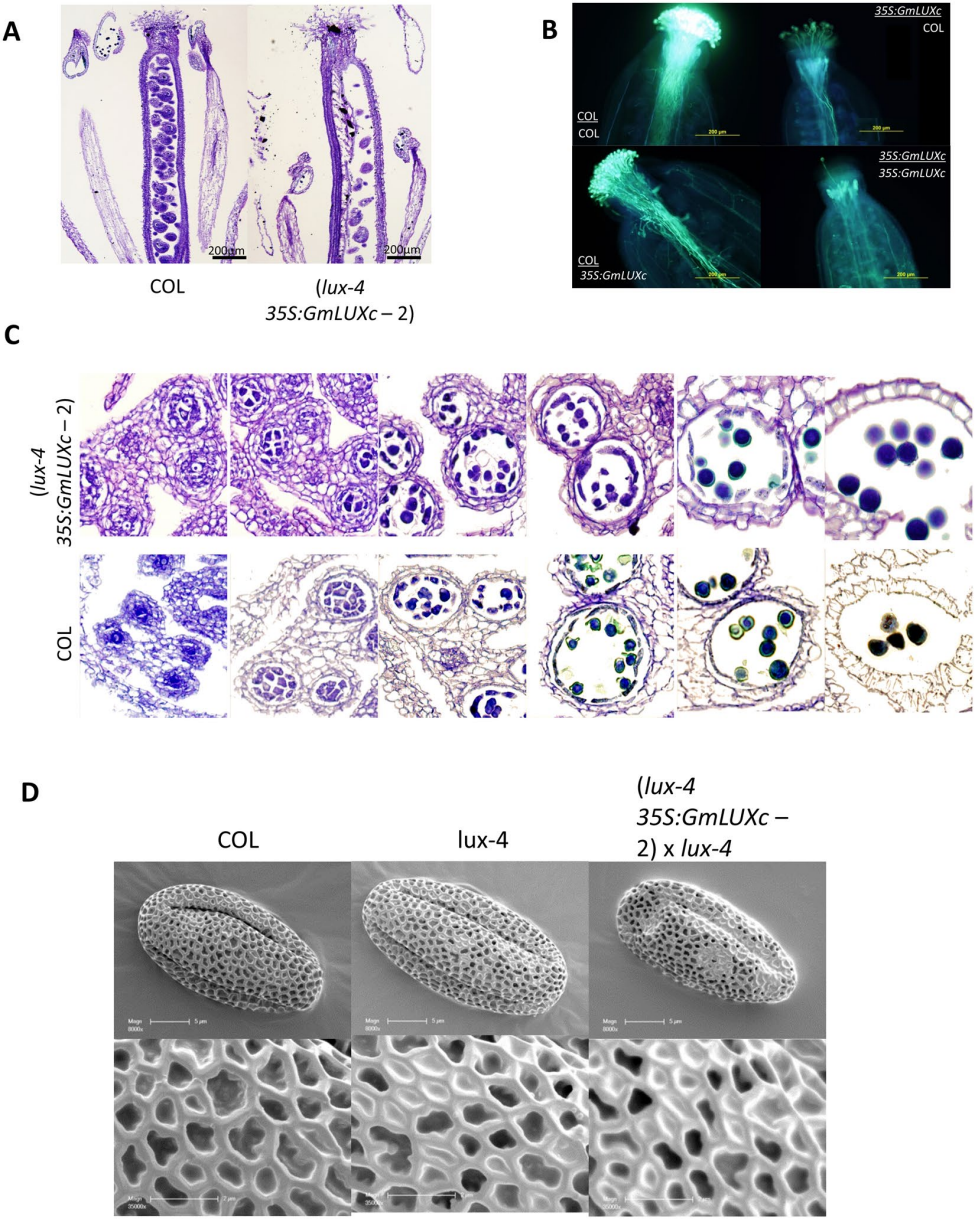
Overexpression of *GmLUXc* in *Arabidopsis* does not affect pollen viability and development. **(A)** Histological sections of Columbia (COL) and *lux-4 35S::GmLUXc* plants showing shorted filaments of *lux-4 35S::GmLUXc* plants. **(B)** *In vivo* pollen germination following reciprocal crossing between Columbia (COL) and *lux-4 35S::GmLUXc* plants. The top genotype is the pollen donor and the bottom genotype is the ovule donor. **(C)** Pollen development of Col wild-type and *35S:GmLUXc lux-4* Arabidopsis plants. **(D)** Scanning EM of pollen from Col, *lux-4* and *lux-4 35S::GmLUXc* plants showing normal pollen exine in all three genotypes.

To further examine male-sterility of overexpression of *35S::GmLUXc* plants, a pollen viability test was performed where all mature pollen from WT and transgenic plants were found viable containing three nuclei, and pollen from WT and transgenic plants looked similar (Fig. 5D). To examine any developmental abnormalities during microsporogenesis, histological analyses on different pollen development stages were carried out. At each stage, pollen development of transgenic plants was indistinguishable from those of wild-type (Fig. 6A,C). Taken together, overexpression of *GmLUXc* does not affect pollen viability or pollen development. We further investigated pollen germination *in vivo*, where self-pollination and cross-pollination of wild-type and overexpression of *35S::GmLUXc* pistils were performed. Analysis of Aniline blue stained pollinated pistils revealed that when *35S::GmLUXc* pistils were pollinated with WT pollen, the pollen tubes growth in pistils was similar to the self-pollinated WT (Fig. 6B). Inversely, pollination of WT pistils with *35S::GmLUXc* pollen grains revealed that the pollen from transgenic plants could not attach effectively to the surfaces of wild-type stigmas and form pollen tubes (Fig. 6B). A similar phenotype was also observed in self-pollinated *35S::GmLUXc* pistils (Fig. 6B). These findings further confirmed that the low seed set of the *35S::GmLUXc* plant is due to male but not female fertility. This *in vivo* pollination and pollen tube germination assay also suggested that *35S::GmLUXc* pollens have difficulty attaching pollen to stigma. We further examined pollen attachment to stigma by evaluating pollen adhesion and pollen retention on the stigma. For pollen attachment, a mature stigma of an emasculated flower was lightly touched three times by freshly dehisced anther, and the pollen grains attached to the stigma surface were observed using a microscope. The pollen of *35S::GmLUXc* transgenic plants failed to attach to the stigma when compared to those from wild-type plants (Fig. 5E, top panel). Pollen retention was also evaluated by washing gently pollinated pistils in sodium phosphate buffer (50 mM, pH 7.0) containing 1% Tween 20. The wild-type and *35S::GmLUXc* pollen retention on stigma was found to be similar (Fig. 5E, bottom panel). From these experiments we concluded that the pollen grains of overexpression of *35S::GmLUXc* plants are defective in pollen adhesion. To examine whether the defect in pollen adhesion is due to any changes in the morphology of pollen exine, we performed scanning EM of pollen. Scanning EM of pollen exine of overexpression *35S::GmLUXc* and WT were found to be indistinguishable (Fig. 6D).

### Gibberellin might be one of the downstream targets of *GmLUXc*

**Figure 7:**
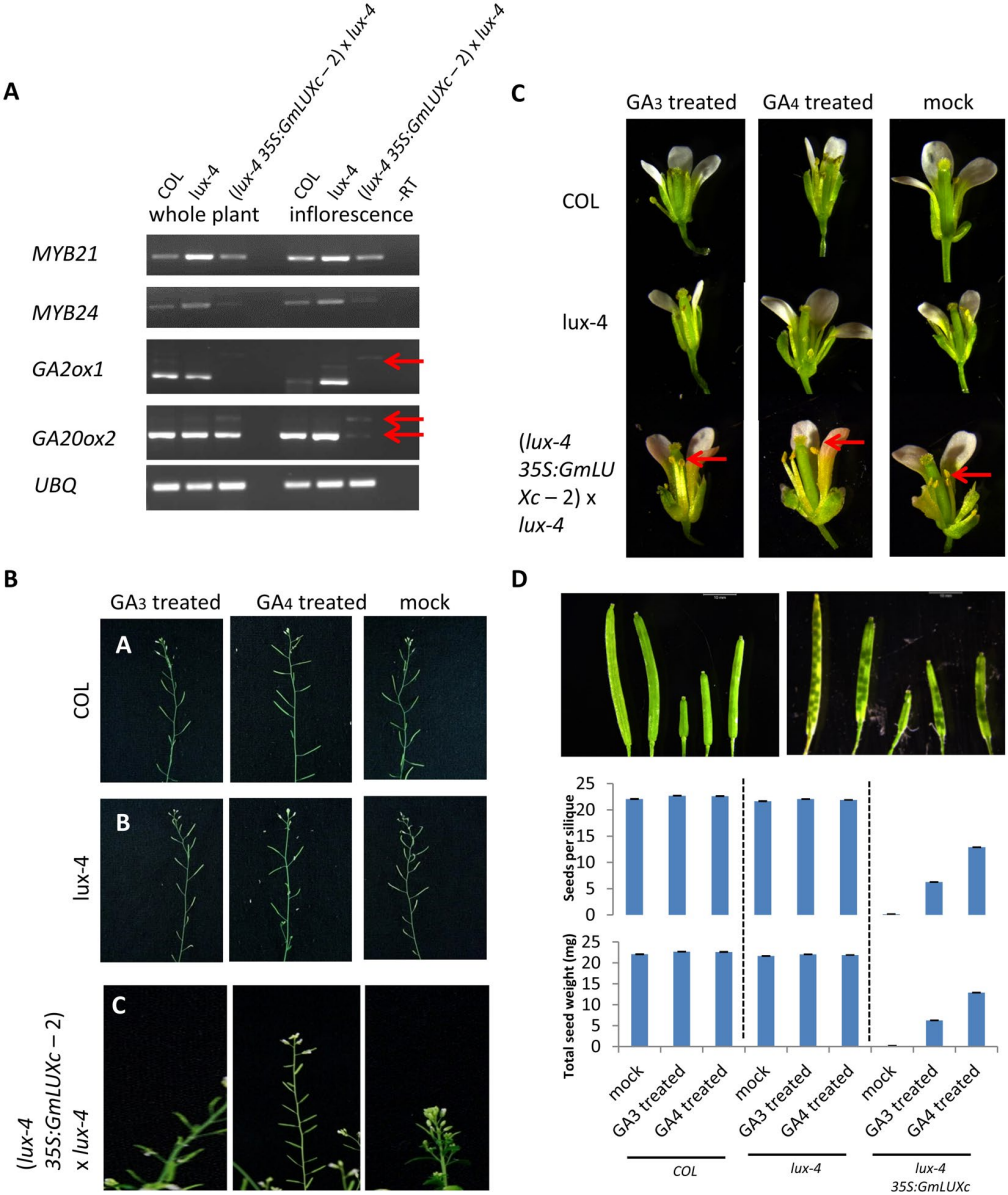
Gibberellin Pathway is one of the downstream targets of GmLUXc. **(A)** RT-PCR of downstream genes shows decrease transcript in *AtMYB21* and *AtMYB24* and splice variants (red arrows) in *AtGA20x1* and *AtGA20ox2*. The data is representative of three biological replicates. Gibberellin (GA_3_ or GA_4_) treatment rescue. **(B)** stunted phenotypes **(C)** filament length (red arrows). **(D)** seed set of *lux-4 35S::GmLUXc* plants. Photos of siliques are from representative plant (from left to right) of *Col*, *lux-4*, *lux-4 35S::GmLUXc* mock, *lux-4 35S::GmLUXc* GA3 treated, and *lux-4 35S::GmLUXc* GA4 treated. Seed set is measured by seeds per siliques and total seed weight. Data are mean ± SE for n = 10–12. Representative plants treated with GA_3_ or GA_4_ or mock (0.02% ethanol) are shown.

To further investigate how *GmLUXc* causes the novel phenotypes, *MYBs* (*MYB21*, *MYB24, MYB57*) genes that are known to affect anther filament length^27^ and genes involved in the jasmonate (*GTR1*, *PDF1*, *DAD1*, *OPR3*), gibberellin (*GA2ox1*, *GA20ox2*), auxin (*RGA*, *GAI*) signalling pathways, were examined in inflorescences and whole *35S::GmLUXc* transgenic plants (Fig. 7A, Supplementary Figure 5). *MYB21*, *MYB24, MYB57* function redundantly in *Arabidopsis* to control stamen filament elongation and mutation in these genes caused reduced filament length in anther^27^. Figure 7A shows that *MYB21* and *MYB24* transcripts were down-regulated in *lux-4 35S::GmLUXc* plants. The GA biosynthesis genes *GA2ox1* and *GA20ox2* showed the presence of splice variants in the *lux-4 35S::GmLUXc* plants (Fig. 7A). Cis-element analysis of the promoter regions of *GmLUXb* and *GmLUXc* revealed higher numbers of GA pyrimidine box ((C/T)CTTTT(C/T)) in *GmLUXc* (10 elements) as compared to *GmLUXb* (4 elements) and *AtLUX* (7 elements) (Supplementary Table 1). These results suggested that *GmLUXc* might regulate those downstream pathways via interacting with the GA signalling pathway. To confirm this hypothesis, exogenous GA_3_ and GA_4_ (dissolved in 0.02% ethanol) were sprayed onto *lux-4 35S::GmLUXc* plants and compared to the *lux-4* mutant and *Col* wild-type. *lux-4 35S::GmLUXc* plants were found to be rescued by GA treatment where plants elongated normally and formed normal inflorescence compared to stunted and compact inflorescence in *lux-4 35S::GmLUXc* plants (Fig. 7B). Examination of anthers in fully opened flowers revealed that the anther length of *lux-4 35S::GmLUXc* plants were rescued by GA treatment (Fig. 7C). Siliques were found to be more normal in size with some properly developed seeds (Fig. 7D). GA_4_ was able to rescue the seed set better than GA_3_ treatment, as indicated by the number and seed weight (Fig. 7D). Almost double the amount of the seeds per silique and total seed weight was obtained from the plants sprayed with GA_4_. Resulting seeds were found to be 100% viable.

## Discussion

The circadian clock is a crucial regulator of different biological functions such as enhances adaptation, fitness, and survival of plants^28–30^. In *Arabidopsis*, the circadian clock system consists of three main interlocking loops: morning, central, and evening loops. The evening complex, a tripartite protein complex of ELF4-ELF3-LUX, is an integral to the plant circadian clock for maintaining circadian rhythms^17, 31^. The evening complex component seems to be positively selected during genome duplication events^21^. Soybean, a palaeopolyploid, has had its genome duplicated several times. The genome sequence of soybean indicated the presence of three soybean homologues of *LUX*. Among the three *GmLUX* genes, *GmLUXa* transcripts were undetectable in all of the tissues examined in this study. Further, sequence analysis indicated that *GmLUXa* is truncated in the middle of the MYB DNA-binding domain, resulting in loss-of-function, suggesting *GmLUXa* is a pseudogene. On the other hand, both *GmLUXb* and *GmLUXc* transcripts were detected in all tissues examined with a high expression in the flower. Although our study clearly shows that *GmLUXb* and *GmLUXc* transcripts are entrained by LD/SD and constant light to display diurnal and circadian rhythms, there is a clear difference in the patterns of circadian rhythms of *GmLUXb* and *GmLUXc* soon after the release of entrained plants into the constant light. Under constant light, *GmLUXb* mRNA was increased gradually, whereas *GmLUXc* mRNA was damped 3-fold of the expression level when compared with the peak under entrained condition, suggesting these might have an additional role at light gating or light sensing. This is the first indication that the three homologues of *LUX* might function differently in soybean where *GmLUXa* becomes a pseudogene, and *GmLUXb* and *GmLUXc* paralogues could undergo subfunctionalization.

**Figure 8:**
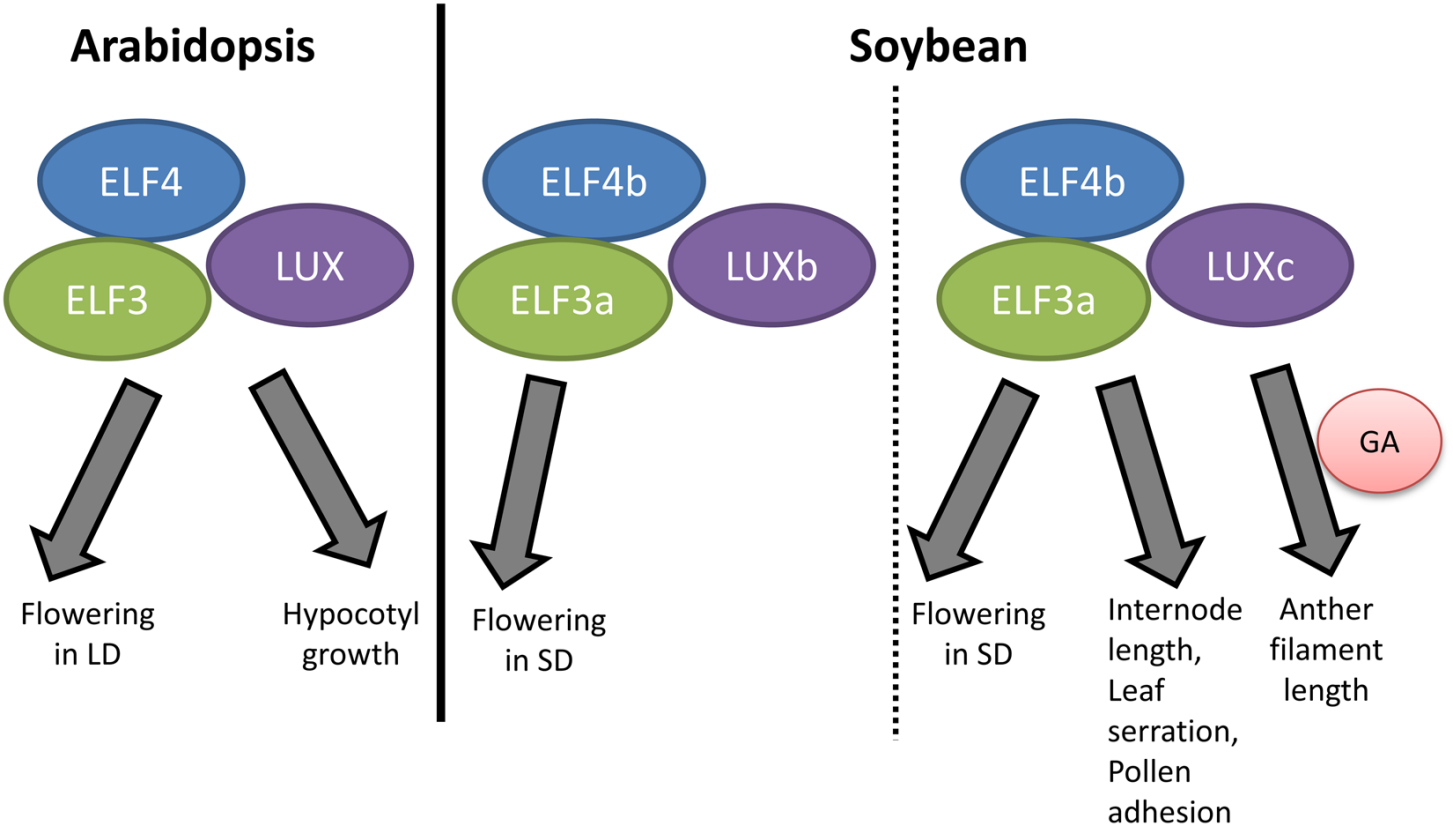
A proposed model for action of soybean LUX paralogues – GmLUXb and GmLUXc. As compared to Arabidopsis, both soybean LUX paralogues, GmLUXb or GmLUXc, form evening complex with GmELF3a and GmELF4b. GmLUXb was shown to only function in flowering time control whereas GmLUXc controls flowering time in SD and also internode length, leaf serration, anther filament length, pollen adhesion. The control of anther filament length may involve Gibberellins (GA).

In *Arabidopsis*, the ELF4-ELF3-LUX evening complex, *ELF4* and *ELF3* encode for novel nuclear proteins, and ELF3 serves as a bridge for the interaction of ELF4 and LUX, while LUX suppress gene expression by recuiting the evening complex to promoters of the target genes^20^ to regulate plant growth^17, 31^. Further, transcripts expression patterns of *ELF4*, *ELF3*, *LUX* are similar, peaking in the evening. To study whether this complex is also conserved in soybean, soybean homologues of *ELF4* and *ELF3* were isolated and characterised. Transcripts of all the four soybean genes—*GmELF4a*, *GmELF4b*, *GmELF3a*, and *GmELF3b*—showed diurnal rhythms, peaking around the same time as *GmLUXb* and *GmLUXc* in the evening under both LD and SD (Fig. 1C). Even orthologs of *ELF3*, *ELF4*, and *LUX*, were found in other species, soybean is the first species other than Arabidopsis shown to form evening complex. Further, our study showed that there are two possible interactions between the three paralogous pairs—ELF4b-ELF3a-LUXb and ELF4b-ELF3a-LUXc—suggesting two putative evening complexes might exist in soybean. In *Arabidopsis*, only one evening complex was present, which controls both flowering and hypocotyl growth; however, in soybean, ELF4b-ELF3a-LUXb seems only to control flowering in SD (Fig. 8), while ELF4b-ELF3a-LUXc not only controls flowering but also filament length and pollen adhesion (Fig. 8). Based on the different phenotypes of plants overexpressing *GmLUXb* and *GmLUXc*, it is highly likely that these two complexes regulate different downstream targets. Above all, our study unravels novel functions of *GmLUXc* in plant development such as control of leaf serration, internode elongation, anther filaments elongation, and pollen adhesion.

The LUX gene has been the focus of recent studies on circadian clocks and photoperiod responsiveness^32–34^. In garden peas, one of the earliest discovered photoperiod responsiveness gene, *STERILE NODE* (*SN*), was recently identified as a homologue of *LUX* by a candidate gene approach^35^. *SN* was found to play role in both the circadian clock and photoperiodic flowering control^35^. A lotus homologue of *LUX* has also been shown to display circadian rhythm under continuous light^36^. In cereals, homologues of *LUX* have been identified as a candidate gene for the early-flowering mutant in barley (*early maturity 10*, *eam10*) and wheat (*earlines per se 3*, *eps-3A*) through fine-mapping^32–34^. Both mutants are early-flowering, photoperiod insensitive, and have distorted diurnal and circadian rhythms for clock genes. Interesting, *LUX* genes in both cereal plants interact with another major determinant of photoperiod response, *Ppd-H1/Ppd* gene, to regulate florigen *FT* (*Flowering Locus T*) expression and regulate flowering time.

Here, we identified *GmLUXb* as the orthologue for *Arabidopsis LUX*, as the *Atlux-4* mutant can be complemented by overexpression of *GmLUXb* to wild-type phenotype with delayed flowering in both LD and SD and reduced petiole length. Intriguingly, *GmLUXc* was found to be neofunctionalised where ectopic expression of *GmLUXc* in *Arabidopsis* causes novel phenotypes of serrated leaves, compact and stunted plants, shortened filament, reduced pollen adhesion, and low seed set. These phenotypes are much more severe under LD where transgenic plants are very small and hardly produce any siliques containing viable seeds followed by early abscission of petals and anthers. Moreover, these phenotypes are correlated with high expression levels of *GmLUXc* in the flower. In garden peas, *SN* showed interaction with another clock gene, *GIGANTEA* (*GI*)/*LATE FLOWERING* (*LATE1*), in regulating flowering node and a total number of reproductive nodes^37^, while wheat *LUX* (*HvLUX1*) sequences are highly correlated with wheat accessions from warmer climates^33^. The results from these studies suggest that molecular functions of the *LUX* gene might have diverged and resulted in novel functions during evolution and domestication of crop plants.

Overexpression of *GmLUXc* produced interesting phenotypes specifically on male reproduction – that is, anther filament elongation and pollen adhesion. This is the first report of circadian clock gene involvement in male reproductive development. Our results showed shortened anther filament length and reduced pollen adhesion in overexpressed *35S::GmLUXc* plants. These findings are consistent with the strong expression of *GmLUXc* in flowers. Moreover, *GmLUXc* expression in mature soybean pollen has been reported previously using soybean Genechip^38^. The underlying mechanism of pollen adhesion has been less studied in the literature. Temperature and the pollen exine coating appeared to be the two major main causes for defective pollen adhesion^39–42^. High-temperature treatment in rice, cherry, and also peach was found to reduce the number of pollen grains that can adhere to stigma^40–42^. A genetic screen for pollen-stigma adhesion mutant in *Arabidopsis* has successfully identified several *lap* (*less adherent pollen*) mutants that have a defective exine layer, suggesting exine is necessary for pollen adhesion^39^. The circadian clock can be entrained by external cues; in addition to light, the temperature is a major input cue^43, 44^. The morning clock component, *PSEUDORESPONSE REGULATOR 7* (*PRR7*) and *PRR9*, have been shown to be important for temperature entrainment in *Arabidopsis*, and the double mutant has impaired sensitivity to temperature signals^45^. Furthermore, a bHLH transcription factor PHYTOCHROME INTERACTING FACTOR4 (PIF4) has been shown to regulate hypocotyl growth and thermal activation of flowering^17, 46^. Function of the EC is essential for regulated expression of *PIF4*, since it represses *PIF4* expression early in the night^17^. Based on these studies on *Arabidopsis*, one might speculate that overexpression of *GmLUXc* might have affected the temperature entrainment of the circadian clock or exine development of pollen, which indirectly affects the pollen-stigma adhesion process and produces a male sterile phenotype. A close observation of the pollen exine of *GmLUXc* and WT plants by scanning EM fail to show any differences.

LUX belongs to an MYB-related GARP family, and its role in anther development has not been reported, though functions of some MYB transcription factors have been reported in anther development including MYB80/103 in pollen and tapetum development^47, 48^ MYB21, 24 in filament elongation^49^ and MYB26 in anther dehiscence^50^. Further, transcriptome analyses of whole Arabidopsis plants have revealed circadian clock regulation of many plant hormone biosynthesis, signalling, and responsive genes^51–53^. In particular, abscisic acid, gibberellin, auxin, and jasmonate have been shown to be targets of circadian outputs^54–56^. Reciprocally, hormones including abscisic acid and cytokinin can act as an input for the circadian clock in Arabidopsis^57^. Moreover, phytohormones including auxin, gibberellin, and jasmonate have been shown to regulate stamen filament elongation. Short filaments were noticed in mutants defective in auxin biosynthetic genes (YUC2, YUC6), auxin response factor (ARF6, ARF8), and auxin transport (MDR1, PGP1)^58^. On the other hand, gibberellin and jasmonate-deficient mutants, *ga1-3* and *opr3*, are both male sterile owing arrested filament elongation, delayed anther dehiscence, and reduced pollen viability^59–61^. Data on Arabidopsis also showed that gibberellin promotes jasmonate biosynthesis influencing *MYB21*, *MYB24*, and *MYB57* expression to control stamen development^62, 63^.

Accordingly, we further investigated whether overexpression of the *GmLUXc* phenotype is due to perturbation of hormones biosynthetic pathways. Indeed, our study found decreased transcript levels of *MYB21* and *MYB24* and gibberellin biosynthesis genes (*GA2ox1*, *GA20ox2*) in *35S::GmLUXc* transgenic plants, but no change on jasmonate and auxin biosynthesis and signalling genes were observed (Supplementary Figure 5). Application of exogenous GA rescued the seed set in *35S::GmLUXc* transgenic plants, suggesting GA could be a downstream target of GmLUXc.

It is now well established that circadian clock gene alleles have played a vital role in the domestication of crop plants by affecting key agricultural traits such as flowering time and yield^62^. Recent research also emphasized the role of the circadian clock in abiotic and biotic stresses responses. Examples of clock genes that have contributed towards adaptation of crop plants include *Pseudo-Response REGULATORS*, *GIGANTEA*, and evening complex genes *ELF3*, *ELF4*, and *LUX*. Preuss *et al*., reported an increase in soybean crop productivity by modulation the expression of morning clock genes^64^. These investigators used *AtBBX32*, B-box domain gene from *Arabidopsis* to change the expression of morning clock genes (*GmTOC1* and *GmLCL2*) transcripts to increase soybean grain yield^64^. Further, it was proposed that the increase in circadian clock genes expression during the transition from dark to light is critical for the reproductive development of soybean^64^.

Given the genomic context of crop plants such as soybean, it is possible that the evening complex genes can regulate multiple outputs as reported in our present study. Thus, an understanding of how clock genes function in crop plants has the potential to uncover new genetic targets for breeding crops that are resilient to climate change.

## Methods

### Plant materials and growth conditions

All plants were grown in a growth cabinet with controlled environments and received nutrient solution weekly. *Arabidopsis* were grown in a 1:3 mixture of perlite and potting mix at 20 °C under either long-day (LD; 16 h light, 8 h dark) or short-day (SD; 10 h light, 14 h dark) conditions under 150 µmol∙m^−2^ s^−1^ white light from cool-white fluorescent tubes. Soybean plants (*Glycine max* L. Merr. cv. Bragg) were grown in potting mix topped with seed raising mix (Debco) at 24 °C under LD or SD condition at 24 °C, 400 µmol∙m^−2^ s^−1^ and 70% humidity. Flowering time was measured as a number of days from the day of sowing to the day when the petals of the first flower were visible and total number of rosette leaves and cauline leaves at bolting. Total seed weight (mg) was measured by weighting the harvested seeds of 10 plants, and by counting the number of seeds yielded by ten siliques we estimate a mean number of seeds per silique.

### Phylogenetic analysis

Protein sequences of different plant species were retrieved from NCBI (https://www.ncbi.nlm.nih.gov/) and soybean genome database at Phytozome (https://www.phytozome.net/). Sequences were aligned in the multiple sequences alignment tool CLUSTALX2.0, using Gonnet Protein Weight Matrix with default parameters. The phylogenetic tree was constructed by the Neighbour-Joining algorithm using the MEGA 5.0. The bootstrap consensus tree was inferred from 1000 replicates.

### Gene expression studies

The expression data of *Glycine max* genes were extracted either from Soybean electronic fluorescent pictograph (eFP) browser at the Bio-Array Resource (BAR) (http://bar.utoronto.ca/efpsoybean/cgi-bin/efpWeb.cgi)^65^. All diurnal and circadian rhythms experiments were conducted under LD or SD or constant light (LL; 12 h light:12 h dark) for three weeks conditions. The soybean seedlings were three weeks old at harvest and samples were collected at four h intervals across a 24 h or 72 h period. Three biological replicates were obtained, and each replicate consists of two unifoliate leaves randomly pooled together from two different plants. Total RNAs were then extracted using SV Total RNA Isolation System (Promega) according to manufacturer’s protocol. Reverse transcription was conducted with one µg of total RNA using SuperScript™ III Reverse Transcriptase (Invitrogen) according to manufacturer’s instructions. Quantitative real-time RT-PCR (qRT-PCR) was performed with 1.5 µL cDNA template in a ten µL reaction volume using Brilliant III Ultra-Fast SYBR® Green QPCR Master Mix (Agilent, USA) with the Stratagene Mx3000 P™ System (Agilent, USA). Two technical replicates were performed for each sample. Standards, a no-template control, and an RT-negative sample were included in each run. Primers used in RT-PCR can be found in Supplementary Table 2. RT-PCR was performed with two µL cDNA template in a 25 µL reaction volume using Taq DNA Polymerase, recombinant (Invitrogen). Three biological replicates were performed and analysed on 1.5% agarose gel. Primers for gene expression studies can be found at Supplementary Table 2.

### Subcellular localization of GmLUXb::GFP and GmLUXc::GFP proteins

Cells in epidermal layers of onion bulbs were transformed with pUC18/2x*35S::GFP::GmLUXb*, 2x*35S::GFP::GmLUXc* or 2x*35S::GFP* by particle bombardment. The full-length *GmLUXb* and *GmLUXc* cDNA fragments were amplified using XbaI-LUXb-F and LUX-KpnI-R primers (Supplementary Table 2). GFP fragment was amplified by BamHI-GFP-F and GFP-NS-XbaI-R primers (Supplementary Table 2). Both fragments were cloned into pGEMT-easy (Promega) and digested with XbaI and SpeI and re-ligated. After digestion with BamHI and KpnI, the digested fragment was cloned into pUC18 containing 2xCaMV35S promoter and Nos terminator. Bombardments were performed using the Biolistic PDS-1000/He Particle Delivery System (BioRad). Fluorescent signals were recorded with an Olympus BX60 fluorescence microscope equipped with an Olympus DP70CCD camera system. The same cells were stained with 1 µgml^−1^ of 4′,6-diamidino-2-phenylindole dihydrochloride (DAPI) in PBS and fluorescence was recorded similarly.

### Complementation and ectopic expression studies

*Arabidopsis elf3-1, elf4-1, and lux-4* or Columbia plants were transformed with Agrobacteria harbouring the binary vector pMLBART/*35S::GmLUXb* or *35S::GmLUXc* using the floral dip method. The full-length soybean *ELF3*, *ELF4*, or *LUX* cDNA fragments were amplified specific restriction enzyme overhang primers (Supplementary Table 2). The resulting PCR products were cloned into pRT101 containing CaMV35S promoter and poly-A terminator. NotI restriction sites were introduced by CaMV35S-NotI-F and polyA-NotI-R primers (Supplementary Table 2) and the PCR fragments were cloned into pGEMT easy (Promega) by TA cloning and were subsequently inserted into the binary vector of pMLBART followed by NotI digestion. Plants treated with GA were sprayed with 50 µM GA_3_ or GA_4_ (Sigma, USA) every two days for two weeks started when plants have 3 cM inflorescence.

### Pollen Analysis

Pollen viability test was performed using mature pollen grains or the anthers with Alexander’s stain or double staining with 0.5 µgml^−1^ fluorescein diacetate (FDA) and one µgml^−1^ propidium iodide (PI) (Regan and Moffatt, 1990). To visualise nuclei of mature pollen grains, open flower were placed to 500 uL of DAPI staining solution (0.1 M sodium phosphate. pH7.5, 1 mM EDTA, 0.1% v/v Triton X-100) and vortexed briefly to release the pollen. The tube was centrifuged and resuspended in 40 µL of DAPI staining solution containing one µgml^−1^ DAPI (Park *et al*., 1998). Aniline blue staining of pollen tube was carried out according to Kho and Baer (1968)^66^. Pistils were cut longitudinally 12 h postpollination and fixed in 3:1 ethanol: acetic acid for two h at room temperature, washed with distilled water and softened overnight in NaOH solution (8 M). The pistils were washed in distilled water for one hour in the following day for three times. It was followed by staining in aniline blue solution (0.1% aniline blue in 0.1 M K_2_HPO_4_-KOH buffer, pH 11) for three hours in complete darkness. The stained pistils were placed in a drop of glycerol and carefully squashed under a cover slip and observed with the fluorescence microscope.

To obtain cross and longitudinal section of developing anthers and flowers, wild-type and transgenic inflorescence were fixed in fixed in 4% w/v paraformaldehyde (Sigma, Australia), 4% w/v DMSO (Sigma, Australia) in phosphate-buffered saline (PBS) with vacuum infiltration. After overnight fixation at 4 °C, samples were subjected to a serial of ethanol dehydration and stained with 0.1% w/v Eosin Y. The samples were then subjected to Histoclear (Sigma, Australia) and embedded in paraplast (Sigma, Australia). Embedded tissues were cut at eight-micrometer sections and stained with toluidine blue. Sections were observed with the light microscope.

To determine pollen adhesion, two assays were carried out based on protocol reported previously^67^. The two assays are used to determine pollen attachment and pollen retention. For the attachment assay, out of the six stamens of fully opened flower, one of the four longer stamens was isolated. The anther of this stamen was used to lightly touch a mature stigma of an emasculated flower for three times. For the retention assay, the same procedures of the attachment assay were carried out. 30 minutes after pollination, the pistil was cut and washed gently in sodium phosphate buffer (50 mM, pH 7.0) containing 1% Tween 20. The stigma with style was then removed from the pistil, mounted on a slide with 80% glycerol for microscope observation.

### Yeast two-hybrid and yeast three-hybrid

Yeast two-hybrid was performed using Matchmaker GAL4 yeast two-hybrid system (Clontech, USA) according to manufacturer’s instructions. Constructs were generated by cloning full-length cDNA sequences into the multiple cloning sites of pGBKT7 and PGADT7 plasmids using relevant primers (Supplementary Table 2). Baits were expressed as Gal4 DNA-BD fusion proteins in the pGBKT7 plasmid; preys were expressed as Gal4 AD fusion proteins in pGADT7 vector and both baits and preys were co-transformed into the AH109 yeast strain. Co-transformants were selected on SD-WL (synthetic drop-out media lacking the tryptophan and leucine). Interactions were tested on selective media lacking tryptophan, leucine, and histidine (SD-LWH + x α-gal). Serial 1:10 dilutions were prepared in water, and five μl of each dilution was used to yield one spot. Plates were incubated at 30 °C for three days before scoring and taking photographs. β-galactosidase activity was assayed for LacZ reporter using o-Nitrophenyl-β-galactoside (ONPG) as the substrate in liquid culture according to manufacturer’s instructions (Clontech). This assay was repeated three times. For yeast three-hybrid assay, pBridge plasmid was used instead of pGBKT7 in the Matchmaker Gold Yeast Two-hybrid system (Clontech) where the third protein is expressed in the absence of methionine (M). Both baits and preys were co-transformed into the Y2Hgold yeast strain. Co‐transformants were selected on SD‐WL. Interactions were tested on SD-WL + AbA (Aureobasidin A) and SD-WLM + AbA.

### Accession numbers for phylogenetic tree

At: *Arabidopsis thaliana* AtLUX (NP_001190022), AtBOA (NP_200765) Ps: *Pisum sativum* PsLUX(KJ801796) Mt: *Medicago truncatula* MtLUXlike (Medtr4g064730), Cr: Chlamydomonas reinhardtii He: Helianthus exilis Ee: Euphorbia esula Gm: *Glycine max* GmLUXa (Glyma01g36730), GmLUXb (Glyma12g06406), GmLUXc (Glyma11g14490), Le: Lycopersicon esculentum, Lj: *Lotus japonicas* LjLUX (chr3.CM0792.250.r2.d), Md: Malus domestica Mc: Mesembryanthemum crystallinum Os: Oryza sativa Ppp: Physcomitrella patens Pg: Picea glauca Pta: Pinus taeda Pt: Populus trichocarpa Pp: Prunus persica Rc: Ricinus communis RcLUXlike (XP_002520534.1) St: Solanum tuberosum Tp: Trifolium pratense Vv: Vitis viniferaa.

## Electronic supplementary material

Supplementary Info
