# Supplementary material for: A novel role of the soybean clock gene LUX ARRHYTHMO in male reproductive development
Source: Sci Rep. 2017 Sep 6;7:10605. doi: 10.1038/s41598-017-10823-y (PMC5587693; doi:10.1038/s41598-017-10823-y)
Supplement: Supplementary file 1 — Supplementary Info [file 41598_2017_10823_MOESM1_ESM.pdf]

```
GmLUXc    : MGEEVKTSEYDEE-----------RVMEWEAGLPTANDLTPLSQPLIPPE :  39
GmLUXb    : MGEEVKTSEYDEE-----------RVMEWEMGLPTANDLTPLSQPLIPPE :  39
GmLUXa    : -------------------------------------------------- :   -
PsLUX     : MGEEIR---TDEE-----------RVTEWEKGLPNLHDLTPLSMTLIPPE :  36
MtLUXlike : MGEEVR---TDEE-----------RVTEWEKGLPNLHDLTPLSMALIPPE :  36
LjLUXlike : MGEEVRM--MDEE-----------RIMEWEKGLPSVHDLTPLSQPLIPPE :  37
AtLUX     : MGEEVQMSDY-----DVSGDGD--RVSEWEMGLPSDEDLASLSYSLIPPN :  43
AtBOA     : MGKEVMVSDYGDDDGEDAGGGDEYRIPEWEIGLPNGDDLTPLSQYLVPSI :  50

GmLUXc    : LASAFSILPEPHRTLLDVNRASRNTLSTLRGGGGSVHQAFSSSNNN-HNY :  88
GmLUXb    : LASAFSISPEPHRTLLEVNRASRNTLSTIRGGG-SVHQAFSSNNNNNHHY :  88
GmLUXa    : ------MSLEPHRIVLNVKRGSCNTLSTIRSDR-TMQHAFFSNNNNNQHY :  43
PsLUX     : LASAFSISPEPHRTLIDVNRASRNTLSILRGGG-TNHQTFSSNNEE-IIE :  84
MtLUXlike : LASAFSISPEPHRTLFDVNRASRNTLSLLRSN--SGTITNQINQTM-SCE :  83
LjLUXlike : LATAFSISPEPRRTLLDVNRASRNTLSILRSGG-APLQALSSSDEEGEGG :  86
AtLUX     : LAMAFSITPERSRTIQDVNRASETTLSSLRGGSSGPNTSSSNNNVEEEDR :  93
AtBOA     : LALAFSMIPERSRTIHDVNRASQITLSSLR-----SSTNASSVMEEVVDR :  95

GmLUXc    : DGDGDGGVEEEEDDDDDRDGSG-PDSRKQRKIDCGAAEEADSAVQTETSA : 137
GmLUXb    : DGDGDGG-DEEEYDDADRDGSG-SDSRKQRKIDCGVAEEADSAVRTETSA : 136
GmLUXa    : NGDGDVN-DKEDNDDTDCKDSR-SDSR------------------TETSA :  73
PsLUX     : EED------EEMEEETDRDGSG-SDSRKLRKIDSATEE----ADSAVRT- : 122
MtLUXlike : IED------IEDEEPDRDGSG-SDSRKHRKIDSVIEEVEAEADSAVRT- : 125
LjLUXlike : GGG------GEEDDTTDRDGSGGSGSRKQRKVDCAAEE----ADSAVRTT : 126
AtLUX     : VGS------SSPGSDSKKQKTSNGDGDDGGGVDPDSAMAAEEGDSGTEDL : 137
AtBOA     : VES------SVPGSDPKKQKKS--DGGEAAAVEDS---TAEEGDSGPEDA : 134

                                   MYB domain
GmLUXc    : ERTAVKRPRLVWTPQLHKRFVDVVAHLGIKNAVPKTIMQLMNVEGLTREN : 187
GmLUXb    : ERTAVKRPRLVWTPQLHKRFVDVVAHLGIKNAVPKTIMQLMNVEGLTREN : 186
GmLUXa    : KRTTVKRLQLVWTLQLHKRFVDVVAHLGIKNAVPKTIMQLMNVEGLSL-- : 121
PsLUX     : -ETTLKRPRLVWTPQLHKRFVDVVAHLGIKNAVPKTIMQLMNVEGLTREN : 171
MtLUXlike : -TETIKRPRLVWTPQLHKRFVDVVAHLGIKNAVPKTIMQLMNVEGLTREN : 174
LjLUXlike : DTATLKRPRLVWTPQLHKRFVDVVAHLGIKNAVPKTIMQLMNVEGLTREN : 176
AtLUX     : SGKTLKRPRLVWTPQLHKRFVDVVAHLGIKNAVPKTIMQLMNVEGLTREN : 187
AtBOA     : SGKTSKRPRLVWTPQLHKRFVDVVAHLGIKNAVPKTIMQLMNVEGLTREN : 184

               MYB domain
GmLUXc    : VASHLQKYRLYLKRMQGLSNEGP--SASDQLFASTPVP-QSLHDSAPPSN : 234
GmLUXb    : VASHLQKYRLYLKRMQGLSNEGP--SSSDQLFASTAVP-QSLHDSAPPSA : 233
GmLUXa    : -------------------------------------------------- :   -
PsLUX     : VASHLQKYRLYLKRMQGLSNDAP--SSSDHLFASTPVP-QSLHESASAVN : 218
MtLUXlike : VASHLQKYRLYLKRMQGLSNDAP--SSSDHLFASTPVP-QSLHETASAN- : 220
LjLUXlike : VASHLQKYRLYLKRMQGLSNEGP--SSSDHLFASTPVP-QSLHDSAAAAV : 223
AtLUX     : VASHLQKYRLYLKRMQGLTNEGP--SASDKLFSSTPVPPQSFQDI----- : 230
AtBOA     : VASHLQKYRLYLKRIQGLTTEEDPYSSSDQLFSSTPVPPQSFQD------ : 228

GmLUXc    : HSNGHGHGHGHSNGRGHGHGHGHGHLS------VPMMSMPYPPPLMSMPY : 278
GmLUXb    : HSN------------------GHGHLP------VPMMSMPYPPPMMSMPY : 259
GmLUXa    : -------------------------------------------------- :   -
PsLUX     : S-----------HSQSNGHGNSHSHHS-------VTISMPYPPPPPPMMS : 250
MtLUXlike : ------------HSNSHSHSHSHSHHS-------VTIPMPYPPPPSSMMS : 251
LjLUXlike : AAT--------AHSNSHSQTHSHSHGHGNFFHLPVPIPMPYPPP---MMS : 262
AtLUX     : ----------------GGGGGSSGNVG---------VPIPGAYGTQQMMQ : 255
AtBOA     : ------------------GGGSNGKLG---------VPVP----VPSMVP : 247

GmLUXc    : PP--PMMSGMP--HGHMGIPMPNSSA----------TSAYHPYNMLHQRD : 314
GmLUXb    : PP--PMMSGMPHAHGHMGIPMPNSSA----------TSAYHPYNMLHQRD : 297
GmLUXa    : -------------------------------------------------- :   -
PsLUX     : MP-LLGMP--PHPHGHMGMALHPPSG--------SSSYRSHPFNMMHHRD : 289
MtLUXlike : MPPMIGMG--HHPHGHMGMSLPPPSGG-------SSSYRSHPFNMMHHRD : 292
LjLUXlike : MP-FHGMPHLHHGHGHMGMQ------------------------------ : 281
AtLUX     : MPVYAHHMGMQGYHHQNHNHDPYHQNHRHHHGAGGNGAFESNPYMMQQNK : 305
AtBOA     : IPGYGNQMGMQGYYQQYSNH--------------GN---ESNQYMMQQNK : 280

GmLUXc    : WP---------HLAPNDK-- : 323
GmLUXb    : WP---------HLAPNDK-- : 306
GmLUXa    : -------------------- :   -
PsLUX     : WPPHPHSHSHPHMSPNDSNK : 309
MtLUXlike : WPPNPHSHSHPHMSSNDSNK : 312
LjLUXlike : -------------KKKKKKK : 288
AtLUX     : FGSMASYPSVGGGSANEN-- : 323
AtBOA     : FGTMVTYPSVGGGDVNDK-- : 298
```

Supplementary Figure 1

Amino acids alignment for LUX-Like protein

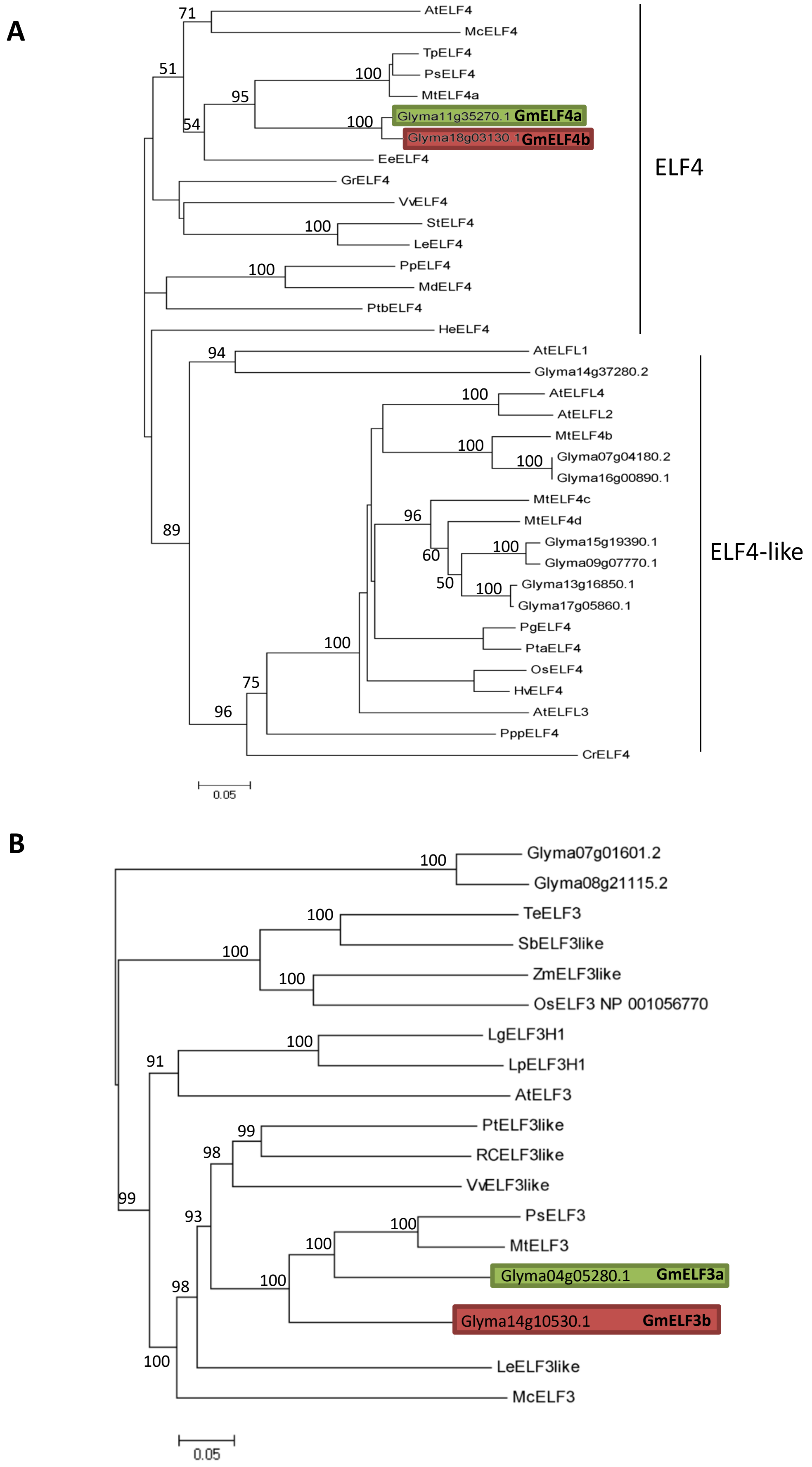

Supplementary Figure 2. Phylogenetic analyses of ELF4-like and ELF3-like proteins.

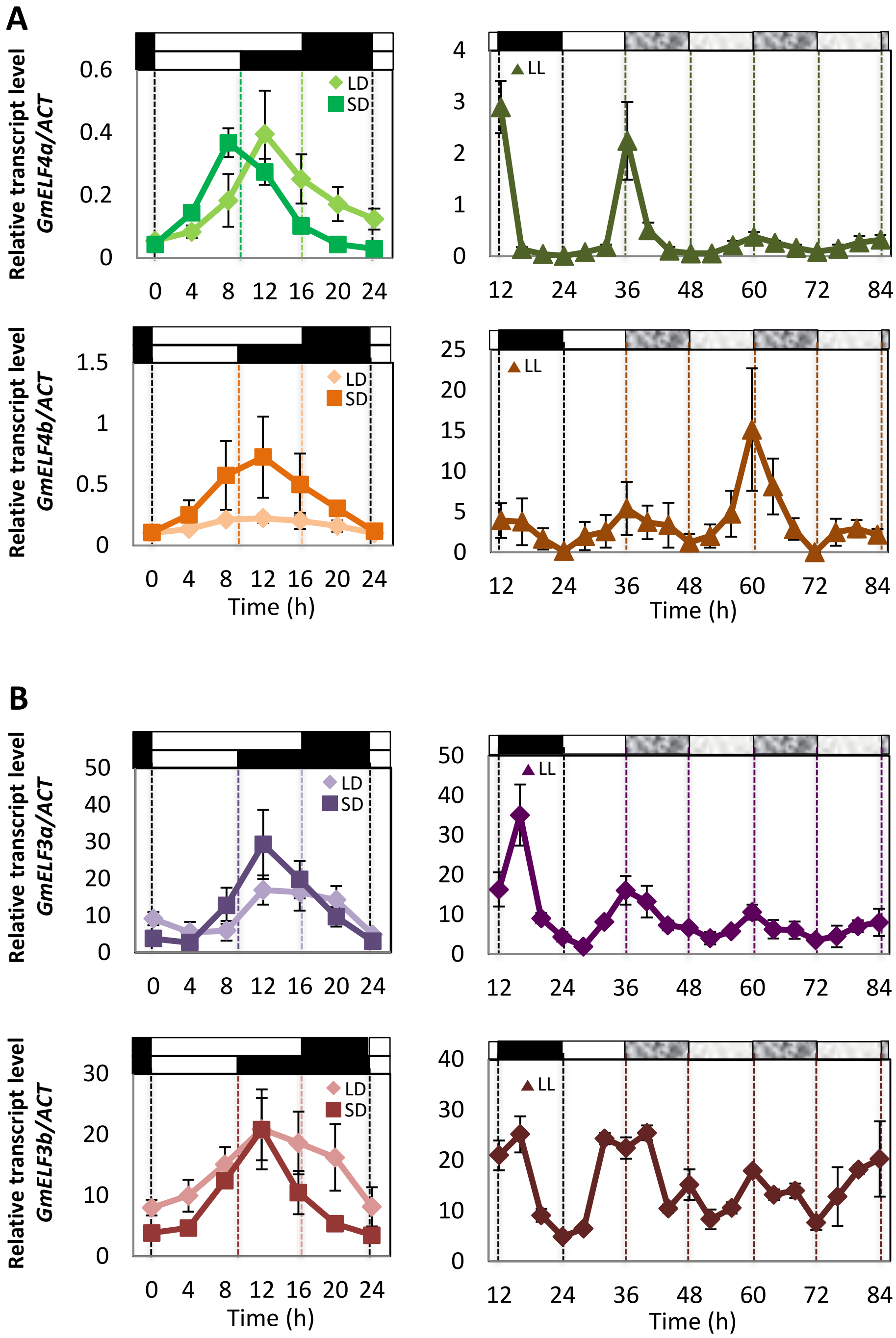

Supplementary Figure 3. **Homologues of soybean *ELF4* and *ELF3* transcripts show diurnal and circadian rhythms.**

(A) *GmELF4a* and *GmELF4b* expression level under long-day long-day (LD; 16h light, 8h dark) or short-day (SD; 10h light, 14h dark), or constant light (LL; 12h light:12h dark then continuous light). **(B)** *GmELF3a* and *GmELF3b* expression level under LD (16L: 8D), SD (10L:14D), or LL (12L: 12D then continuous light). All plants were three weeks old at the time of sampling. Data are mean ± SE for n = three biological replicates, each consisting of pooled material from two plants. Day and night periods are indicated by open and closed bars, respectively, above the graph.

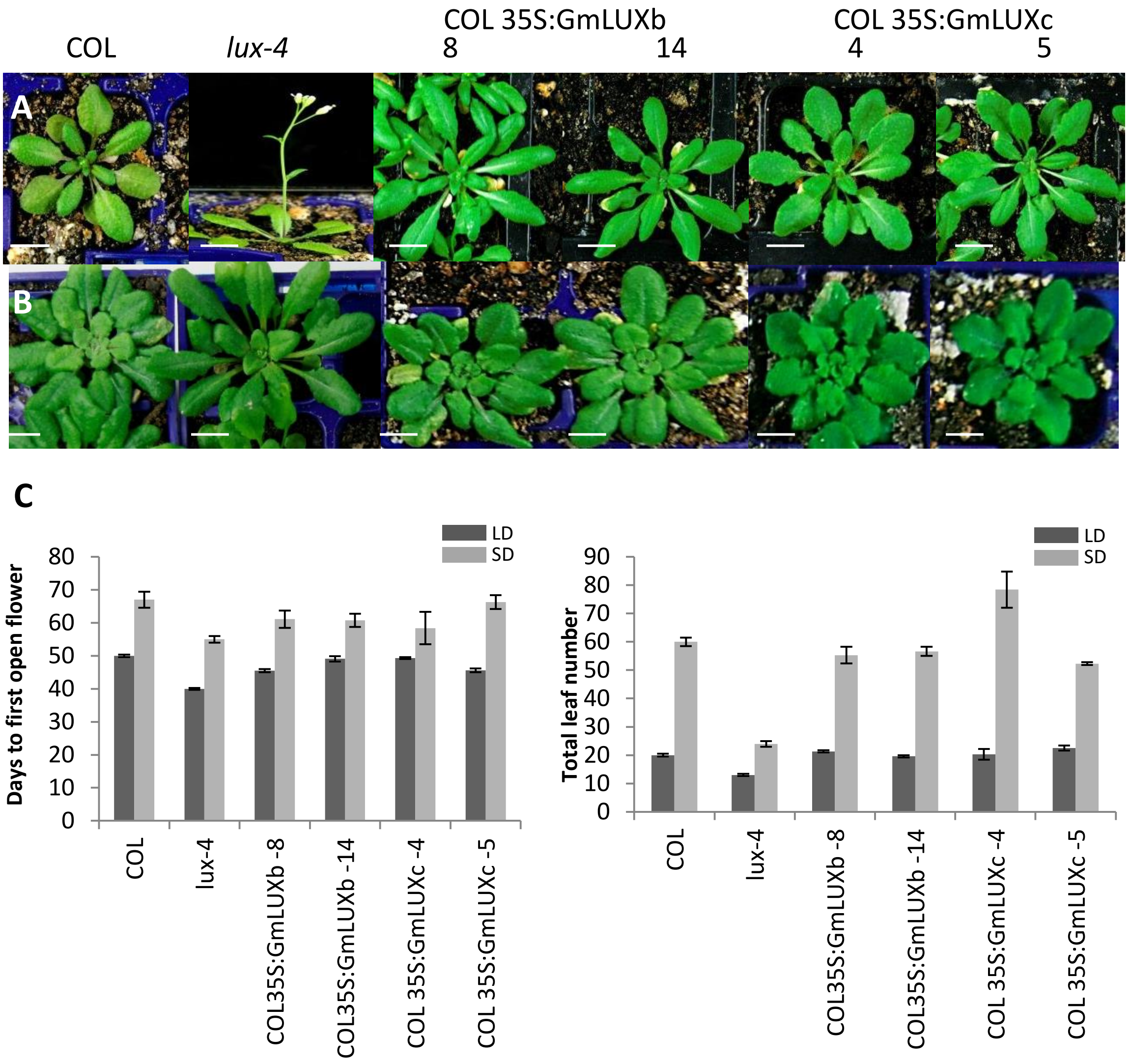

Supplementary Figure 4. Ectopic expression of *GmLUXb* and *GmLUXc* in *Arabidopsis Columbia* (*Col*) show similar phenotype as in *lux-4* mutant.

(A) Representative plants are grown in LD (16L: 8D). **(B)** Representative plants are grown in SD (10L:14D). **(C)** Flowering time of plants grown in LD and SD, data are mean ± SE for n = 15-20.

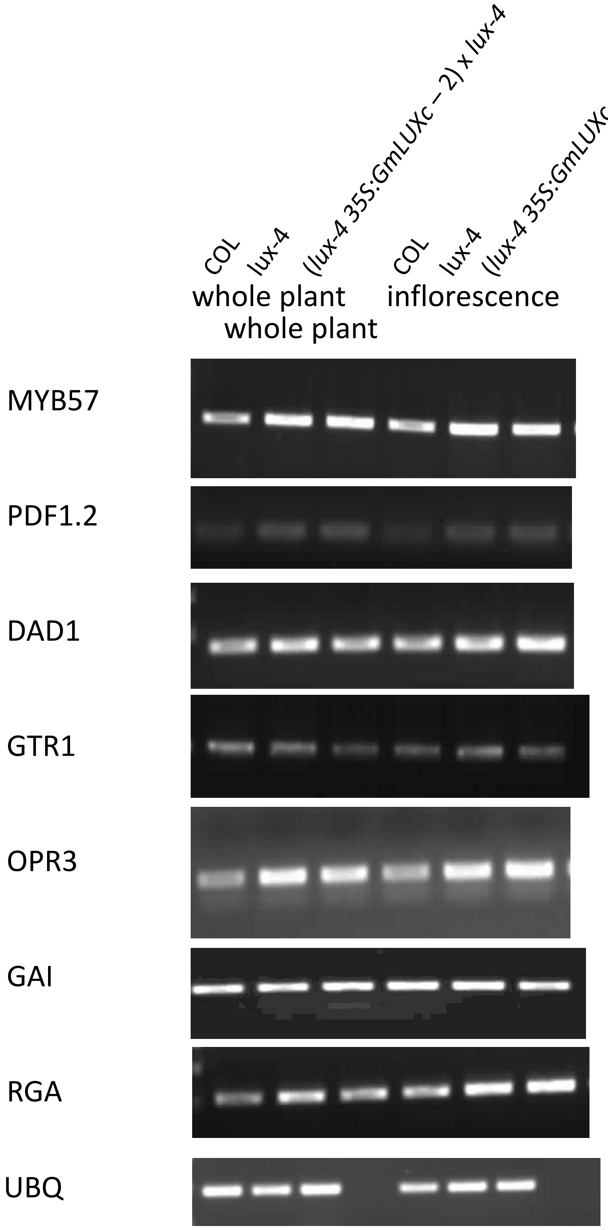

Supplementary Figure 5. Expression of *MYB57*, *GTR1*, *PDF1*, *DAD1*, *OPR3*, *RGA*, *GAI* genes in *35S::GmLUXc* transgenic plants.

Supplementary Table 1. *Cis*-element analysis of 5' regions of *GmLUXb* and *GmLUXc* in comparison to *AtLUX.*

Response	Site Name	Sequence	AtLUX	GmLUXb	GmLUXc
**Circadian**	Clock	CAANNNNATC	3	2	4
	CCA1	AAMAATCT	0	0	1
	Evening element	AAAATATCT	1	1	1
	LBS (LUX binding sites)	GATWCG	2	0	1
**Gibberellin**	TAACA(A/G)A element	TAACA(A/G)A	1	1	1
	TATCCA(C/T) element	TATCCA(C/T)	1	1	0
	pyrimidine box	(C/T)CTTTT(C/T)	7	4	10
**Ethylene**	GCC element	GCCGCC	1	0	0
	A(A/T)TTCAAA element	A(A/T)TTCAAA	1	0	1
**Cytokinin**	cytokinin response motif (CRM)	(A/G)GAT(T/C)	30	16	17
**Auxin**	ABRE	(C/T)ACGTG	3	3	1
	TGTCTC element	TGTCTC	1	1	1
**Light**	I-box	GATAAGR	1	0	1
	G-box	CACGTG	2	2	0
	H-box	ACCTA(A/C)C(A/C)	1	0	0
	Sp1	CC(G/A)CCC	0	1	1
**Sugars**	TATCCA element	TATCCA	1	2	0
	SURE	(AA)TACTA(A/T)T	1	0	2
	W-box	(T)TGAC(C/T)	0	1	1
**Cold**	C-repeat	CCGAC	3	1	0
	LTR	CCGAAA	0	2	1

Supplementary Table 2. Primers sequences used in gene isolation, Real-Time PCR, and construct cloning.

Primer name	Sequence (5'-3')	Uses
ACT-F	ATCATGTTTGAGACCTTCAATGTG	RT-PCR
ACT-R	CTCGAGTTCTTGCTCATAATCTAGG	
LUXb-3F	AATGGCCATGGTCATCTCCC	RT-PCR
LUXb-3R	GCACCTTCTATAACATTGTA	
LUXc-4F	AATGGTCATGGTCATGGGCA	RT-PCR
LUXc-1R	ATGATGGTGATGGCTGACCT	
BamHI-GFP-F	GGATCCATGGTGAGCAAGGGCGAGGAG	pUC18/2x35S::GFP::GmLUXb, 2x35S::GFP::GmLUXc
GFP-NS-XbaI-R	GCTCTAGACTTGTACAGCTCGTCCATG	
LUX-XbaI-F	GCTCTAGAATGGGGGAAGAGC	pUC18/2x35S::GFP::GmLUXb, 2x35S::GFP::GmLUXc
LUX-KpnI-R	CCGGGTACCTCATTTATCATTAG	
LUX-EcoRI-F	CCGGGAATTCATGGGGGAAG	vector pMLBART/35S::GmLUXb or 35S::GmLUXc
LUX-BamHI-R	CGCGGGATCCTCATTTATCA	
CaMV35S-NotI-F	ATTTGCGGCCGCATGGTGGAGCACGAC	pMLBART/35S::GmLUXb or 35S::GmLUXc
polyA-NotI-R	AGGAGCGGCCGCACTGGATTTTGGTTT	
T7 promoter 2	TAATACGACTCACTATAGGGCGA	Yeast-two hybrid cloning
3' BD sequencing 2	AGCAACCTGACCTACAGGAA	
3' AD sequencing	AGATGGTGCACGATGCACAG	
Gal4_N_term_primer	GAGTAGTAACAAAGGTCAA	Yeast-three hybrid cloning
pHybLex_rev_primer	GAGTCACTTTAAAATTTGTATACAC	
AtUBQ10-F	GGCCTTGTATAATCCCTGATGAATAAG	RT-PCR
AtUBQ10-R	AAAGAGATAACAGGAACGGAAACATAGT	
AtGTR1-F	GTCCATTGGCTGGTATTGCT	RT-PCR
AtGTR1-R	ACTTGCTGCAACGTGCATAG	
AtPDF1.2-F	TTTGCTGCTTTCGACGCAC	RT-PCR
AtPDF1.2-R	CGCAAACCCCTGACCATG	
AtDAD1-F	GTGAAGACGAAGAAGAAGAGCAATC	RT-PCR
AtDAD1-R	GTGAAGACAGCGAAAACGACATAC	
AtOPR3-F	TTGGACGCAACTGATTCTGAC	RT-PCR
AtOPR3-R	GTAGGCGTGGTAGCGAGGTT	
AtMYB21-F	AAAATCGCCAAACATCTTCC	RT-PCR
AtMYB21-R	AATTATAACCCCAAACCTCTACAA	
AtMYB24-F	ATGCAAAATGGGGAAATAGGTG	RT-PCR
AtMYB24-R	AAGATCATCGACGCTCCAATAGTT	
AtMYB57-F	GTGCGGCGAGGGAACATAA	RT-PCR
AtMYB57-R	TCAGCAATAGAAAAACCAAATAAC	
AtGA2ox1-F	CGGTTCGGGTCCACTATTTC	RT-PCR
AtGA2ox1-R	ACCTCCCATTTGTCATCACCTG	
AtGA20ox2-F	CCGGCAGAGAAAGAACACGAA	RT-PCR
AtGA20ox2-R	TACGCCTAAACTTAAGCCCAGAA	
AtRGA-F	AGAAGCAATCCAGCAGA	RT-PCR
AtRGA-R	GTGTACTCTCTTCTTACCTTC	
AtGAI-F	CACACGACCGCTCATAG	RT-PCR
AtGAI-R	TGCCTATCCAATTTACCCTC	
